# Anticancer Activities of Mushrooms: A Neglected Source for Drug Discovery

**DOI:** 10.3390/ph15020176

**Published:** 2022-01-31

**Authors:** Sujogya Kumar Panda, Gunanidhi Sahoo, Shasank S. Swain, Walter Luyten

**Affiliations:** 1 Center of Environment, Climate Change and Public Health, RUSA 2.0, Utkal University, Bhubaneswar 751004, India; 2 Department of Zoology, Utkal University, Bhubaneswar 751004, India; gunanidhi.nou@gmail.com; 3 Department of Biology, KU Leuven, 3000 Leuven, Belgium; walter.luyten@kuleuven.be; 4 Division of Microbiology and NCDs, ICMR-Regional Medical Research Centre, Bhubaneswar 751023, India; swain.shasanksekhar86@gmail.com

**Keywords:** anticancer activity, bioactive compounds, clinical trials, in vitro, in vivo, medicinal mushrooms, pharmacological potential

## Abstract

Approximately 270 species of mushrooms have been reported as potentially useful for human health. However, few mushrooms have been studied for bioactive compounds that can be helpful in treating various diseases. Like other natural regimens, the mushroom treatment appears safe, as could be expected from their long culinary and medicinal use. This review aims to provide a critical discussion on clinical trial evidence for mushrooms to treat patients with diverse types of cancer. In addition, the review also highlights the identified bioactive compounds and corresponding mechanisms of action among the explored mushrooms. Furthermore, it also discusses mushrooms with anticancer properties, demonstrated either in vitro and/or in vivo models, which have never been tested in clinical studies. Several mushrooms have been tested in phase I or II clinical trials, mostly for treating breast cancer (18.6%), followed by colorectal (14%) and prostate cancer (11.6%). The majority of clinical studies were carried out with just 3 species: *Lentinula edodes* (22.2%), *Coriolus versicolor*, and *Ganoderma lucidum* (both 13.9%); followed by two other species: *Agaricus bisporus* and *Grifola frondosa* (both 11.1%). Most in vitro cell studies use breast cancer cell lines (43.9%), followed by lung (14%) and colorectal cancer cell lines (13.1%), while most in vivo animal studies are performed in mice tumor models (58.7%). Although 32 species of mushrooms at least show some promise for the treatment of cancer, only 11 species have been tested clinically thus far. Moreover, most clinical studies have investigated fewer numbers of patients, and have been limited to phase III or IV. Therefore, despite the promising preclinical and clinical data publication, more solid scientific efforts are required to clarify the therapeutic value of mushrooms in oncology.

## 1. Introduction

Estimates of the number of fungal species on Earth range widely, from around half a million to 10 million. Recent estimates by Hawksworth and Lucking indicate 2.2–3.8 million from which only 120,000 species have been named so far [1]. Thus, only about 8% of the estimated number of species is presently known to mycologists [2]. The estimated number of mushroom species on Earth is 150,000–160,000 [3]; however, so far, only ~14,000 species are identified, of which ~7000 have varying degrees of edibility, with 3000 species mainly edible and falling within 21 genera [4]. Moreover, ~2000 species are estimated to be useful medicinally, while only 270 species are reported to possess therapeutic potential for human health [4]. Therefore, mushrooms have already proved themselves as a potential source of drugs against both communicable and non-communicable diseases based on clinical reports. In addition, they supplement primary food in daily life and contain several unique secondary metabolites, polysaccharides, essential minerals, proteins, and vitamins [5,6]. However, only 10% of existing mushroom species are known to science so far, while <1% is exploited for therapeutic uses. From this perspective, mushrooms appear to be a neglected natural source, whose therapeutic potential deserves to be explored in a scientific manner for the discovery of new drugs.

At present, cancer is a leading cause of death worldwide. Nearly 10 million deaths were recorded globally from different cancers in 2020 [https://www.who.int/news-room/fact-sheets/detail/cancer; assessed on 1 January 2022]. Cancer arises through several external factors or agents, such as physical carcinogens by ultraviolet or ionizing radiation, chemical carcinogens by the consumption of contaminated water, food, transition metals, asbestos, aflatoxin, tobacco smoke, etc., and biological carcinogens, such as certain viruses, bacteria, and parasites. It arises through a transformation of normal cells into tumor cells by a multi-stage process from a pre-cancerous lesion to a malignant tumor. According to WHO reports, in 2020, 2.2 million cases of breast cancer were recorded, leading to 685,000 deaths, 2.21 million cases of lung cancer were recorded, with 1.18 million deaths, 1.93 million cases of colon and rectum cancer were recorded, with 935,000 deaths, and 1.09 million cases of stomach cancer were recorded, with 769,000 deaths. “Despite the growing success of conventional personalized cancer therapies, recurrence and metastases remain common, depending on the type of cancer and the stage of disease” [6]. Although early detection and appropriate diagnosis play a crucial role in cancer management, the development of anticancer treatments through synthetic chemicals, or by exploring unique metabolites extracted from mushrooms or other natural sources, is a promising approach to help clinical oncology in developing new cancer drugs.

Over 60% of anticancer drugs can be traced to a natural products, but none so far originate from a mushroom [7]. This is surprising, since mushrooms have long been claimed to have anticancer effects. Traditionally, mushrooms have been used for treating cancers. “Over the past three decades, scientific and medical research in Japan, China, and Korea and recently in the USA have confirmed the properties and unique compounds extracted of mushrooms for prevention and treatment of cancer and other chronic diseases” [8]. A few of these species include: *Agaricus, Albatrellus*, *Antrodia*, *Calvatia*, *Clitocybe*, *Cordyceps*, *Flammulina*, *Fomes, Funlia*, *Ganoderma, Inocybe*, *Inonotus*, *Lactarius*, *Phellinus*, *Pleurotus*, *Russula*, *Schizophyllum*, *Suillus*, *Trametes*, and *Xerocomus*, etc. They show promising anticancer activity and may contain potent anticancer compounds. Dunneram et al. have suggested inclusionding of more mushrooms in our diet as a protective measure against cancer [5]. As such, the market for dietary supplements containing mushrooms is rapidly growing, with a market size of over 18 billion USD. This represents about 10% of the overall market for dietary supplements. Fungal genera, such as *Ganoderma, Ophiocordyceps*, and *Cordyceps*, have a prominent share [6].

Mushroom-derived polysaccharides exhibit potent antitumor activity against several tumor metastasis cells. Moreover, they showed better activity when used in conjunction with chemotherapy. Mechanistically, the antitumor action is facilitated through a thymus-dependent immune mechanism, which necessitates an intact T cell component. Polysaccharides class components mainly trigger cytotoxic macrophages, natural killer cells, dendritic cells, monocytes, neutrophils, and chemical messengers that activate complementary and acute phase responses. In addition, these polysaccharides act as multi-cytokine inducers, capable of stimulating gene expression of many immunomodulating cytokines and their receptors [7,8,9,10]. Terpenes are another class of compounds, well known for their bioactivity, and many mushroom-derived terpenes have shown potential anticancer properties. Terpenes can modulate the immune system by inducing the expression of genes coding for proteins engaged in the immune response. Mushrooms are also a rich source of carbohydrate-binding proteins known as lectins, and exhibit cytotoxicity/anticancer properties with different mechanisms of action. Several lectins are known to have antitumor and antiproliferative properties. Other important metabolites include phenolic compounds, well known as antioxidants with different mechanisms of action. “Overall, mushroom treatment in oncology studies appeared safe and devoid of side effects. Changes in chemical parameters or clinical signs suggest that mushrooms do affect body physiology, but clinical benefits were more on quality of life than on hard endpoints like disease-free survival or mortality” [9]. 

The present review aims to provide a critical discussion on the clinical trial evidence for mushrooms that can be used in the treatment of diverse types of cancer. In addition, the review also highlights the best-known mushrooms with their identified bioactive compounds and corresponding mechanisms of action. Furthermore, it also discusses the mushrooms with anticancer properties either in vitro or in animal models, which have not yet been tested in clinical studies.

## 2. Summary Results of Literature Analysis

Types and stages of cancer, study parameters (such as sample sizes, dose, treatment duration), and outcomes were noted for each trial with a particular mushroom species (Supplementary Materials, Tables S1–S3). The literature search was performed in PubMed, combining the terms “mushroom” and “cancer”, and limiting the results to clinical trials (https://pubmed.ncbi.nlm.nih.gov/?term=mushrooms+and+cancer&filter=pubt.clinicaltrial&filter=pubt.randomizedcontrolledtrial; assessed on 21 September 2021). This yielded clinical trials on the following medicinal mushrooms (MM): *Agaricus bisporus* (single trials, [10]); *A. blazei* (three trials, [11,12,13]); *A. sylvaticus* (two trials; [14,15]); *Antrodia cinnamomea* (single trial, [16]); *Coriolus versicolor* (two trials; [17,18]); *Ganoderma lucidum* (single trial, [19]); *Grifola frondosa* (three trials; [20,21,22]); *Lentinus edodes* (four trials; [23,24,25,26]); *Phellinus rimosus* (single trial; [27]); *Poria cocos* (single trial; [28]) (Supplementary Materials, Table S1). 

In parallel, we searched clinical trial databases to record clinical trial information and added some additional trials on *Agaricus bisporus* (one randomized phase II trial for prostate cancer, NCT04519879; one interventional clinical trial for breast cancer, NCT007090200), *Coriolus versicolor* (one randomized, parallel, double-blind, placebo-controlled trial for breast cancer, NCT00647075), *Grifola frondosa* (one randomized, interventional clinical trial for lung neoplasms and breast carcinoma, NCT02603016), and *Trametes versicolor* (one phase I clinical trial for breast cancer, NCT02568787) (Supplementary Materials, Table S3). Interestingly, four published reports on *Agaricus blazei* were based on one trial, but presented different results [16,18,29,30].

**Figure 1:**
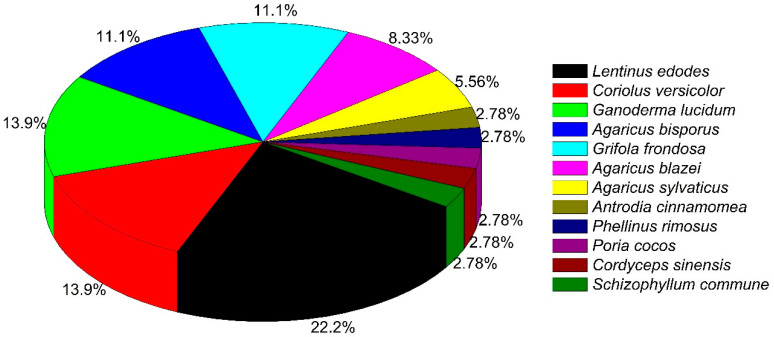
Distribution of mushroom species used in different clinical trials.

We found that the majority of clinical studies were carried out with just 3 species: *Lentinula edodes* (22.2%), *Coriolus versicolor*, and *Ganoderma lucidum* (both 13.9%); followed by *Agaricus bisporus* and *Grifola frondosa* (both 11.1%) (Figure 1). There were 2 other species of *Agaricus* which were also well studied, including *A. blazei* (8.3%) and *A. sylvaticus* (5.6%). Most clinical studies were conducted in humans, except one in dogs (whose results are not impressive, as Maitake^@^ treatment could not reduce lymph node size by more than 50%, while two dogs developed adverse effects [21] (Supplementary Materials, Table S1)).

**Figure 2:**
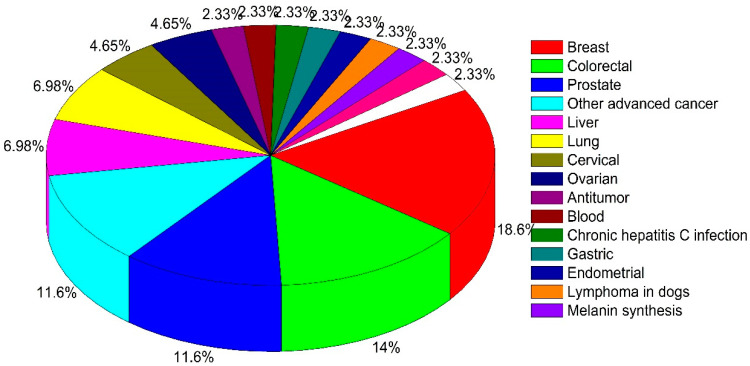
Distribution of various type of cancer among clinical trials.

The distribution of different types of cancer across the reviewed clinical studies is shown in Figure 2. Most studies were treating breast cancer (18.6%), followed by colorectal (14%) and prostate cancer (11.6%) (Figure 2). Other treated cancer conditions included liver, lung (both 6.98%), cervical, and ovarian cancer (both 4.65%) (Figure 2). Few of these studies were placebo-controlled, double-blind randomized trials (RCTs). The administration of mushrooms was largely oral. Several clinical trials studied a combination with chemotherapy to reduce side effects and improve quality of life (QOL), and observe changes in hematologic parameters (HP), overall survival (OS), antitumor activity, or immunomodulation.

**Figure 3:**
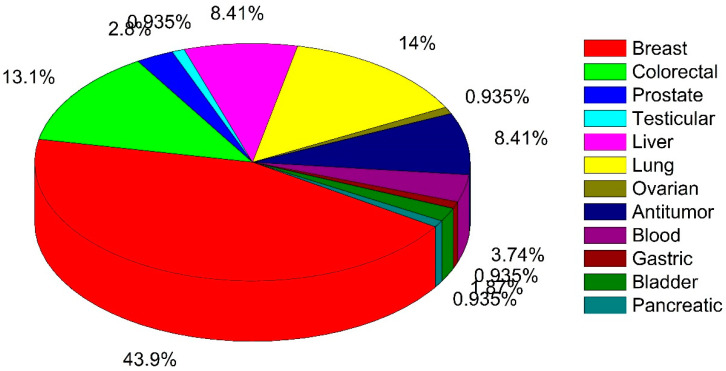
Distribution of various type of cancer among in vitro anticancer studies of mushrooms.

**Figure 4:**
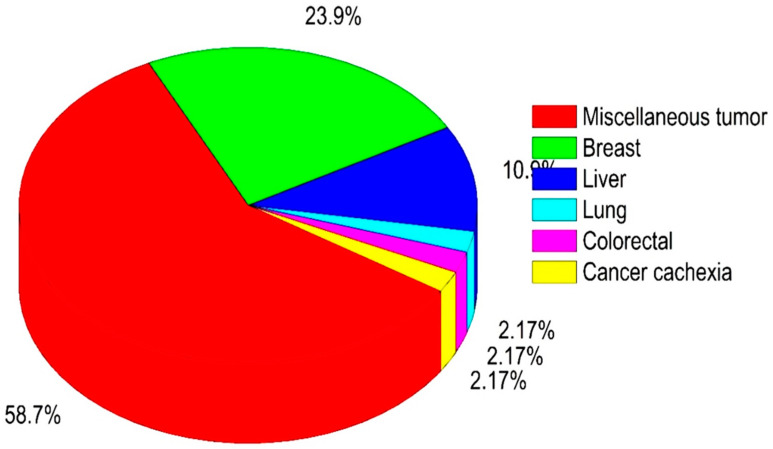
Distribution of in vivo anticancer studies for various type of cancer.

As only a few mushroom species (11) were evaluated in clinical studies, and the total number of studies was small (only 36), we continued our literature search for finding preclinical oncology evidence on mushroom species (database source: https://pubmed.ncbi.nlm.nih.gov/; assessed on 11 October 2021). The anticancer properties from these are summarized for individual species including the type of extracts/fraction/active compounds, type of cancer study, in vitro/in vivo, the dose of treatment, the mechanism involved, etc. (see Supplementary Material Tables S4 and S5). Figure 3 and Figure 4 show the distribution of mushrooms with various cancer types for in vitro and in vivo studies, respectively. The most interesting clinical trials with their major outcomes are summarized in Table 1. For more details and further information, see Supplementary Tables S1–S3.

For in vitro studies with cell lines, most used breast cancer cell lines (43.9%), followed by lung (14%) and colorectal (13.1%), as well as (8.41%) liver cancer cell lines (Figure 3). For in vivo studies, most papers pertained to antitumor effects in mice (58.7%), which may be due to the common availability of tumor mouse models (Figure 4). Moreover, among the cancer types once more breast cancer is most studied (23.9%) followed by liver cancer (10.9%) (Figure 4). To facilitate interpretation, we summarized all mushrooms species per type of cancer in Table 2, listing in vitro vs. in vivo as well as clinical trials.

For more detailed interpretation, a summary table (see Supplementary Information Tables S4 and S5) was prepared listing the name of the mushroom species aimed at different types of cancer. After analyzing the types of studies, each category, such as in vitro, in vivo, in silico, isolation of active constituents, and clinical study, was rated with scores—ideal (excellent, >5 studies-***), (good, 3–5 studies-**), (poor, 1–2 studies-*), or no study (-)—and the overall strength of recommendation score was given based on the available literature (Table 3). 

## 3. Clinical Trials for Various Cancer Types

### 3.1. Treatment of Breast Cancer

Dietary supplementation with *A. sylvaticus* (2.1 g/d for a period of 6 months) for 46 stage II and III breast cancer patients receiving chemotherapy in a randomized, placebo-controlled, and double-blind clinical trial, showed an improved appetite and comparatively fewer gastrointestinal problems, nausea symptoms, and vomiting symptoms [19] (Supplementary Materials, Table S1). Simultaneously, 80% of patients in the placebo group suffered from LOA and gastrointestinal problems, such as diarrhea, constipation, and vomiting, whereas only two treated patients reported similar complaints. 

The spores of *Ganoderma lucidum* are a popular nutraceutical, and have been used to reduce breast cancer-related fatigue and improved QOL [19]. A 4-week treatment of 48 breast cancer patients under endocrine therapy (RCT), with powdered spores of *G. lucidum* considerably (*p* < 0.01) improved QOL compared with the placebo. Several parameters, namely fatigue (week 4, verum = 46.78 ± 5.07; placebo = 40.92 ± 5.62), sleep disturbance (week 4, verum = 42.3 ± 26.2; placebo = 53.9 ± 24.8), and LOA (week 4, verum = 24.3 ± 18.4; placebo = 30.3 ± 16.5) were significantly improved (*p* < 0.01, *p* < 0.01 and *p* < 0.05, respectively). In addition, appetite (week 4, verum = 4.1 ± 2.9; placebo = 6.1 ± 3.2) and depression (week 4, verum = 3.1 ± 2.8; placebo = 4.6 ± 2.9) were significantly improved (*p* < 0.05 and *p* < 0.01, respectively) compared with the control group. Mild discomforts such as dizziness (16%) and dry mouth (12%) were recorded in the verum group [19] (Supplementary Materials, Table S1).

Deng et al. [20] examined the major functional changes in response to oral intake of *G. frondosa* polysaccharide extracts (5–7 mg/kg daily) in 34 postmenopausal breast cancer patients, who became disease-free after primary treatment as a part of phase I/II trial. They observed increases in TNF-α, IL-2, and IL-10 production, but about a one-fifth reduction in IFN-γ production (Supplementary Materials, Table S1). 

Administration of freeze-dried mycelial powder of *Trametes versicolor* to 11 female cancer patients (Phase I, 6–9 mg/d) [18] resulted in an enhancement of lymphocyte counts and NK cell functional activity, in addition to an upsurge in CD8(+) T cells and CD19(+) B cells, but not CD4(+) T cells (Supplementary Materials, Table S1). Yamaguchi et al. [26] combined *Lentinula edodes* with chemotherapy and found this to be safe, with improvements in the QOL of gastrointestinal (two) and breast (three) cancer patients (Supplementary Materials, Table S1). However, drawing conclusions from studies with such a small number of patients is difficult.

### 3.2. Treatment of Lung Cancer 

In an RCT (*n* = 68), *G. lucidum* extracts significantly increased the Karnofsky scores (by >10 in 50% of verum patients compared with 14% (*n* = 29) in the placebo group) in advanced-stage lung cancer patients. In the verum group, 28% of patients (9) had unchanged, and 22% (7) had reduced Karnofsky scores, compared with 46% (13) and 39% (11), respectively, in the placebo group [34]. In addition, a significant improvement (43–84% of treated patients) was observed in the frequency of symptoms such as fever, cough, sweating, weakness, and sleeplessness compared with placebo (11–43%). Furthermore, most of the immune parameters either remained unaffected or were reduced in the control group. A significant improvement was also observed in the mitogenic reactivity of lymphocytes, percentage of CD3, and activity of NK cells, as well as a marginal enhancement in CD4 and a reduction in CD8 to concanavalin A treatment. Treatment of lung cancer patients with Ganopoly^®®^ (*G. lucidum*) in an open-label trial enhanced their immune responses [33,36] (Supplementary Materials, Table S2).

### 3.3. Treatment of Colon Cancer

A group of 56 colorectal cancer patients after surgery was randomly assigned to the administration of *Agaricus sylvaticus* or placebo over a period of 6 months [37]. Although the verum group did not show significant differences in QOL, it registered a tendency toward improved mood and sleep, reduced gastrointestinal discomforts and pain, along with encouraging hematological and glycemic effects [37] (Supplementary Materials, Table S1). The verum group registered a considerable within-group decrease in fasting plasma glucose, cholesterol, creatinine, and several other HP after 3 and 6 months of treatment. However, the weight and body mass index remained unchanged. 

A multi-institutional randomized prospective protocol developed by Nakano et al. [38] on the effect of lentinan in conjunction with other chemotherapeutic agents among advanced-stage gastric cancer patients survival and QOL (Supplementary Materials, Table S2). Another multi-center clinical study involving 80 advanced-stage patients revealed the improvement of colorectal cancer patients reported considerable improvement in QOL scores after 12 weeks of SDL administration [39] (Supplementary Materials, Table S2). Zuo et al. [35] observed a significant improvement in the symptoms of Qi and Yin deficiency in 60 patients receiving Yunzhi glycopeptide. A decrease in the number and size of adenomas was also reported for colorectal adenoma patients (*n* = 198), treated with *Ganoderma lucidum* extract (1.5 g/d).

### 3.4. Treatment of Liver Cancer

Patients with advanced hepatocellular carcinoma (HCC) (*n* = 15) with a liver malfunction, treated (RCT) with *Coriolus versicolor*, had longer median OS compared with placebo (6.5 vs. 2.2 months, respectively, as well as longer median progression-free survival (2.5 vs. 1.1 months) [17] (Supplementary Materials, Table S1). Additionally, treated patients had lower IL-17F and MCP-1 and higher prolactin and TNF-related apoptosis-inducing ligands. Overall, treated patients suffered fewer gastrointestinal side effects and diarrhea compared with placebo. Grinde et al. [11] also observed changes in mRNA (qPCR) in a clinical trial with chronic hepatitis patients receiving β-glucan extract from *Agaricus blazei* (Supplementary Materials, Table S1).

In an RCT (*n* = 78 patients, 136 tumors), patients underwent transcatheter arterial chemoembolization and radiofrequency ablation, and were subsequently treated with lentinan 500 mg/d for 18 months. The treatment increased the average survival period as well as tumor necrosis and reduced the relapse rate in HCC [40] (Supplementary Materials, Table S2). In another multi-center study (*n* = 36 out of 40 HCC patients), survival of HCC patients increased when treated with food supplemented with SDL [41] (Supplementary Materials, Table S2).

### 3.5. Treatment of Leukemia or Blood Cancer

Generally, trials of leukemia patients with mushrooms are not encouraging. Griessmayr et al. [21] treated dogs with lymphoma (*n* = 13) with *Grifola frondose* extract (Maitake^@^) but did not observe a reduction in the size of the lymph node by more than 50%. Hematological parameters, including electrolytes and hepatic and renal values, remained normal throughout the treatment, but two dogs developed hyphema (adverse effects). A phase II trial in blood cancer patients (maitake powder, 3 mg/kg twice daily for 12 weeks) recorded beneficial immunomodulatory potential in myelodysplastic syndromes (MDS) [26] (Supplementary Materials, Table S1).

### 3.6. Treatment of Prostate Cancer

A study with *Agaricus bisporus* powder (6 dosages starting with 4 g/d, and the maximum dosage capped at 14 g/d) in prostate cancer patients (*n* = 32) included several parameters such as evaluation of toxicity, effect on serum PSA/androgen levels and cytokine levels, etc. The extract appeared to reduce prostate cancer by reducing immunosuppressive factors [10] (Supplementary Materials, Table S1). However, studies conducted by DeVere White et al. [25] and Sumiyoshi et al. [24] with *Lentinus edodes* extract failed to detect any significant effect on prostate cancer (Supplementary Materials, Table S1).

### 3.7. Treatment of Gynecological Cancer

Ahn et al. [13], in an RCT (*n* = 100) involving gynecological (cervical, endometrial, and ovarian) cancers under chemotherapy, registered progress with *Agaricus blazeii* in mood parameters and body strength compared with controls. Moreover, mushroom-treated patients had fewer side effects, such as alopecia, LOA, emotional instability, and general weakness. The activity of natural killer cells was significantly enhanced in the treated group after 3 and 6 weeks, compared with placebo, without any significant difference in WBC, monocytes, lymphocytes, T cells, a cluster of differentiation (CD) 48+, and CD 56+ cells, etc. (Supplementary Materials, Table S1).

### 3.8. Treatment of Miscellaneous Cancers and Meta-Analyses Study

Tsai et al. [16] administered *Antrodia cinnamomea* in an RCT (*n* = 37) including breast, lungs, stomach, liver, and colorectal cancer patients receiving chemotherapy, and showed significant improvement in sleep. In addition, most hematological, liver, and kidney functions did not alter significantly, while a significant reduction in platelet cell count (*p* = 0.02) was recorded during a 30-day treatment period (Supplementary Materials, Table S1). 

*Lentinus edodes*, (3 g/d of AHCC^®®^ p.o.,) [23] significantly improved the QOL scores of cancer patients, and decreased levels of herpes virus in saliva during chemotherapy, without hematotoxicity and hepatotoxicity (Supplementary Materials, Table S1). Oral administration of *Ganoderma* capsules (Wuse-Lingzhi—Jiaonang) in a randomized, controlled trial (*n* = 72) improved the functioning of the immune system in radiotherapy-treated nasopharyngeal cancer cases but did not reduce the side effects of radiotherapy [42] (Supplementary Materials, Table S2).

Oba et al. [43] found that lentinan (main ingredient of *Lentinula edodes*) significantly extended the OS (stratified log-rank *p* = 0.011) with an overall hazard ratio (HR) of 0.80 (95% confidence interval = 0.68–0.95) without heterogeneity between trials. The effect of lentinan was probably more effective in lymph node metastasis compared with non-node metastasis patients (*p* for interaction = 0.077). For survival of advanced gastric cancer patients, lentinan, along with regular chemotherapy, has a significant advantage over chemotherapy alone. In an observational case–control study among ovarian cancer patients (*n*= 500), intake of white bottom mushrooms appeared to be indirectly related to the occurrence of epithelial ovarian cancer [44] (Supplementary Materials, Table S2). Okamura et al. [34] studied the effects of polysaccharides from *Schizophyllum commune* in cervical cancer patients (stage II or III, *n* = 220) monitoring several parameters, such as tumor response, time of recurrence, survival, immunologic parameters, and side effects. They could not find any significant change in the survival rate of patients with stage III cancer, but SPG increased the survival time in stage II patients (Supplementary Materials, Table S2). Another meta-analysis (3117 patients from 38 RCTs) in China concluded that the overall response rate in lung cancer treated with lentinan (1–1.5 mg/d, 2–8 weeks) was increased from 43.3% (chemotherapy alone) to 56.9% for chemotherapy plus lentinan (pooled response rate 0.79, 95% CI: 0.74–0.85) [45]. 

Eliza et al. [46] observed that treatment with *C. versicolor* reduced the 5-year mortality of cancer patients up to 9%, particularly in case of breast, colorectal, and gastric cancer patients undergoing chemotherapy. 

In summary, there is a slow but steady surge in the use of mushrooms and their products in modern medicine. Most mushroom derived products are yet to undergo rigorous evaluation following standard protocols of evidence-based medicine like that of synthetic drugs. Consequently, clinical studies, both in animals and human volunteers (healthy or with specific diseases/conditions), are a very important step in introducing novel drugs to the market. However, most of the studies we found are either clinical trials or observational studies; although, a few meta-analyses were also carried out [43,46].

The clinical trials need to be designed, executed, and analyzed aiming for maximal reproducibility. Ideally, the trials should be randomized, double-blind, and placebo-controlled; whereas, many studies we found were single-blind or open-label trials without placebo. Although it is expected that the outcomes of clinical trials may vary based on chosen measurements, treatment/observation duration, extent, and cost, most studies were small, and generally only extended to phase I or II. Although a properly designed observational study is accomplished of providing objective and statistically substantial information, confirmation is required by a randomized controlled trial.

## 4. Preclinical Evidence (Selected Important In Vitro vs. In Vivo Studies)

Linoleic acid-conjugated ingredients of *Agaricus bisporus* suppressed testosterone-induced cell proliferation in MCF-7aro cancer cells, but did not show any activity against non-tumorigenic MCF10A cells. The extract also inhibited tumor growth in nude mice bearing MCF-7aro xenografts [47]. 

*Amauroderma rude* retarded cell survival and initiated apoptosis in MDA-MB-231 breast cancer cells. Tumor growth in athymic nude mice bearing MDA-MB-231 xenografts was also reduced, and the death of tumor cells was stimulated by *A. rude* treatment. Jiao et al. [48] have reported the downregulation of expression of the c-Myc oncogene. In another study, *A. rude* derived ergosterol suppressed the viability of breast cancer cells through apoptosis and upregulation of the expression of the tumor suppressor Foxo3 [49]. Pan et al. [50] isolated a polysaccharide F212 from *A. rude* that increased macrophage metabolism, the proliferation of lymphocytes and the in vivo production of antibodies in tumor growth.

A fermented culture broth of *A. camphorata* downregulated matrix metalloproteinase-2 and -9, urokinase plasminogen activator and its receptor, vascular endothelial growth factor, and the phosphorylation of related proteins. At the same time, the tissue inhibitors of these pathways were upregulated, followed by the arrest of the cell cycle and apoptosis [51]. A submerged fermentation culture of *A. camphorata* induced cell cycle arrest at G1, DNA fragmentation, ROS (reactive oxygen species) production, dysfunction of mitochondria and Bcl-2/Bax, and apoptosis; it also downregulated cyclin D1, PI3K/Akt, and downstream effectors β-catenin and GSK-3β [52]. Antrocia, an *A. camphorate* derived steroid and a known Akt/MTOR dual inhibitor, was found to be a potential candidate for clinical trials against metastatic breast cancer [53]. It hinders multiplication of metastatic breast cancer MDA-MB-231 cells and phosphorylation of Akt; it downregulates Bcl-2, Bcl-xL, and survivin expression; and upregulates expression of cytosolic cytochrome c and Bax, which promotes apoptosis.

The proliferation of MCF-7 cells and tamoxifen-resistant MCF-7 cells is suppressed by ethanolic extracts of *A. cinnamomea*. This extract further showed higher antiproliferative activity toward tamoxifen-resistant MCF-7 cells when administered with tamoxifen [54]. *Antrodia salmonea* extract induced cytoprotective autophagy and apoptosis through extracellular signal-regulated kinase (ERK) signaling cascades [55]. Antcin-A has been reported to reduce the migratory and invading tendency of breast cancer cells [56]. Qiao et al. [57] have reported the presence of several triterpenoids and 8 bio-transformed metabolites in the plasma of rats dosed with *A. cinnamomea*. Ergostanes appeared to be the major plasma-exposed constituents of *A. cinnamomea*, which were generally absorbed and eliminated rapidly, unlike lanostanes. 

*A. salmonea* downregulates the levels of cyclin A, B1, E, and CDC2 proteins, thereby arresting MDA-MB-231 cancer cells at G2 phase of the cell cycle. Besides, suppression of tumor incidence, growth, and migration in athymic nude mice bearing MDA-MB231 xenografts was also observed [58]. In addition, the morphological alterations and epithelial-to-mesenchymal transition through the suppression of *N*-cadherin, nail, vimentin, Twist, and Slug, and enhancement of E-cadherin was reported [59]. 

MycoPhyto^®®^ Complex (*Agaricus blazei*, *Cordyceps sinensis*, *Coriolus versicolor*, *Ganoderma lucidum*, *Grifola frondosa* and *Polyporus umbellatus*, plus *Saccharomyces cerevisiae*-derived β-1,3-glucan) is a dietary supplement [60]. It arrested the highly invasive MDAMB-231 human breast cancer cells at the G2/M phase of the cell cycle through downregulation of cell cycle-regulating genes. Human breast cancer cells treated with cordycepin (3-deoxyadenosine) derived from *C. sinensis* showed reduced cell viability and cellular proliferation, increased cellular release of lactate dehydrogenase and reactive oxygen species, and nuclear apoptosis [61]. Antiapoptotic proteins, such as Bcl-2, were downregulated, while pro-apoptotic proteins, e.g., Bax, caspase-3, 8, and 9, were upregulated. Nude mice with MCF-7 xenograft showed slower tumor growth. Coriolus versicolor extract exhibited antiproliferative activity in T-47D, MCF-7, and MDA-MB-231 cells, and enhanced nucleosome development [62]. The migration and invasion of 4T1 breast cancer cells was blocked by an aqueous extract of *C. versicolor* [63]. Besides, tumor necrosis factor-α, interferon-γ, interleukin-2, 6, and 12 were downregulated in xenograft-bearing mice.

Cordycepin and zhankuic acid A, isolated from *Antrodia cinnamomea*, proved effective against human lung adenocarcinoma through MAPK and PI3K/AKT signaling pathways [64]. *Cordyceps sinensis*-derived cordycepin blocks ADP-induced platelet aggregation, and prevents hematogenic metastasis in B16-F1 mouse melanoma cells [65]. Cordycepin also inhibits non-small cell lung cancer cell cycle progression [66]. In H1975 cells, cordycepin also inhibited cell proliferation and promoted apoptosis via the EGFR signaling pathway [61]. 

Cell migration, tumor growth, and the epithelial mesenchymal transition in breast cancer was prevented by a fucose-containing fraction of *G. lucidum* (FFLZ). The synergistic activity of FFLZ and trastuzumab reduced resistance to trastuzumab [67]. The proliferation of large mammary tumors from MDAMB-231 cells was slowed down, along with reduction in cell migration after one-month oral administration of *G. lucidum* extract. Wu et al. [68] observed a reduction in c-Myc, cyclin D1, CDK2, CDK6, and pRb; induction of DNA fragmentation and PARP cleavage; disruption of mitochondrial membrane potential; and G1 phase cell arrest in DM MCF-7 cells treated with ganoderic acid. The polysaccharides of *G. frondosa* increased the release of lactate dehydrogenase, accumulation of ROS, and elicited mitochondrial dysfunction among others in MCF7 and MDA-MB-231 breast cancer cells [69].

Maitake d-fraction has been reported to reduce the size of mammary, hepatic, and pulmonary cancers in patients receiving chemotherapy and immunotherapy simultaneously. The fraction alone blocked metastasis, downregulated tumor marker expression, and improved NK cell activity [70]. It altered the expression of genes involved in stimulation of multidrug sensitivity, cell cycle arrest, inhibition of cell growth and proliferation, apoptosis, suppression of migration, and metastasis [71]. Moreover, Maitake d-fraction boosted cell–cell adhesion through the upregulation of E-cadherin protein levels, β-catenin membrane localization, and cell–substrate adhesion. In addition, this fraction also delayed tumor growth and shortened pulmonary metastases in a murine model bearing tumor xenografts [72]. Besides, Pro4X, a Maitake d-fraction, reduced angiogenesis, carcinogenesis, invasiveness, and prolonged survival in BALB/c mice bearing breast tumor xenografts [73].

## 5. Toxicity Observations and Lack of Effect in Clinical Trials

Adverse events (AE) following treatment with mushrooms are generally not mentioned explicitly, except in a few trials [16,19,29]. This needs to be interpreted with caution, since some of the AE could be due to the underlying disease or concurrent treatment. Placebo-controlled groups and double-blind evaluation are therefore necessary for proper interpretation of AE. Breast cancer patients under endocrine therapy along with *G. lucidum* recorded mild discomfort such as dizziness (16%) and dry mouth (12%) [19]. Mycelial extracts of *Lentinula* failed to reduce by 50% or more the prostate specific antigen levels in a phase II study of 74 expectantly managed early stage prostate cancer patients [24]. Besides, DeVere White et al. [25] noted the failure of shiitake mushroom extract to lower the prostate-specific antigen levels or even keep them stable in 62 prostate cancer patients.

Fortes et al. [37] followed 56 post-surgery colorectal cancer patients for 6 months while they were treated with *Agaricus sylvaticus* extract, but did not find any significant improvements in QOL between treatment and placebo groups [37]. In an RCT with 37 lung, breast, liver, stomach, and colorectal advanced adenocarcinoma patients undergoing chemotherapy for 30 d, combined with *Antrodia cinnamomea* or placebo, the verum group showed no significant improvements other than sleep (*p* = 0.04) [16]. More frequent but less intense (grade 1 and 2) gastrointestinal symptoms (abdominal pain and diarrhea) were reported for the treated group due to disease progression. Several hematological, kidney, or liver functions, and mean OS did not differ significantly between the two groups. Advanced adenocarcinoma patients showed no significant alteration in OS compared with the controls. Oka et al. [29] reported AE (diarrhea—four patients; stomach discomfort—one patient; poor health—one patient) in 6 out of 123 colorectal adenoma cases receiving *G. lucidum*.

## 6. Mushroom-Derived Active Components and Related Clinical Trials

Several mushroom-derived components show direct antitumor activity and prevent oncogenesis and metastasis. Polysaccharides substantially improve cancer-related symptoms when used in combination with chemotherapy. Such polysaccharides induce gene expression of several immunomodulating cytokines and their receptors [69,74,75]. β-glucan, a mushroom-derived glucose polymer, stimulates NK cells, neutrophils, monocytes, macrophages, and T cells and manifests immunomodulatory and antiproliferative effects [70,76,77,78,79,80,81,82,83]. Schizophyllan, a β-d-glucan isolated from *Schizophyllum commune*, combined with tamoxifen, decreased the incidence of breast tumors, and initiated apoptosis in hepatic carcinomas [84].

Lectins, ergosterol, ganodermanontriol, ganoderic acid, and some of their derivatives have important roles in cancer therapy. Both animal in vivo and human clinical studies support lectins as therapeutic agents. In tumors, they can initiate cytotoxicity, apoptosis, induce cell cycle arrest, downregulate telomerase activity, block angiogenesis, and inhibit tumor growth through preferential binding to cancer cell membranes. They bind to ribosomes and obstruct protein synthesis through the alteration of production of several interleukins and activation of protein kinases [85,86,87,88,89].

Ergosterol or provitamin D2 plays an important role in vitamin D biosynthesis. Its presence is reported in *Agaricus* and it demonstrated antitumor and antiproliferation effects in several cancer cells [82,90]. It also prevents angiogenesis, but has no direct in vitro cytotoxicity [83]. Ergosterol, ergosterol peroxide (5α,8α-epidioxy-22 ergosta-6,22-dien-3β-ol), and 5,6-dehydroergosterol extracted from *G. lucidum* demonstrated in vitro antiproliferative activities. Ergosterol peroxide is well documented for its anticancer properties in breast cancer cells, by arresting the cell cycle (G1 phase), activating caspase-3/7, and PARP cleavage. Furthermore, it attenuated the expression of total AKT1, AKT2, BCL-XL, cyclin D1, and c-Myc in inflammatory breast cancer cells. Ergosterol peroxide sulfonamide exhibited high-level potency in inflammatory breast cancer cells [91].

Zhu et al. [92] demonstrated that ganodermanontriol 24S, 25R)-24,25,26-trihydroxylanosta 7,9(11)-dien-3-one isolated from *G. lucidum* inhibited colony formation and proliferation of MDA-MB-231 cells. In addition, several invasive behaviors, cell adhesion, and cell migration were also inhibited in this breast cancer cell line. 

Ganoderic acid Me (*G. lucidum*) downregulated the expression of NF-κB-regulated genes, including cyclin D1, c-Myc, Bcl-2, matrix metalloproteinase-9, etc., in MDA-MB-231 cells [93]. Another compound, ganoderic acid A (*G. lucidum*), increased the production of ROS, downregulated phosphorylation of JAK2, and inhibited STAT3 downstream activation, as well as gene expression [45]. Out of several mycophenolic acid derivatives, 6-((2E, 6E)-3,7,11-trimethyldedoca-2,6,10-trienyl)-5,7-dihydroxy-4-methylphtanlan-1-one and eburicoic acid from cultured *L. sulphureus* showed moderate cytotoxicity [94,95] but the mechanism of action was not studied.

Panepoxydone, the fermentation product of *L. crinitus*, inhibited I kappa B α 9 (an inhibitor of NF-κB) phosphorylation, and kept the NF-kappa B complex inactive [96]. Panepoxydone exerted anti-proliferative activity on MCF-7, MDA-MB-231, 468, and 453 breast cancer cells. Attenuation of invasion and migration and triggering of apoptosis was also observed in these cell lines. Expression of cleaved Bax and PARP was amplified, but that of Bcl-2, caspase-3, cyclin D1, and cell survival was reduced [97]. Schweinitzins A and (S)-torosachrysone-8-O-methyl ether, two major constituents in the methanolic extracts of *X. schweinitzii* fruiting bodies, showed potent anticancer activity against several types of human cancer cells such as breast, liver, lung, and epidermal cancer [98]. A 66-kDa laccase from *Tricholoma mongolicum* with N-terminal amino acid sequence GIGPVADLYVGNRI, is effective against breast cancer MCF7 cells with an IC_50_ of 4.2 μM [99].

The dietary supplement BreastDefend, a combination of several mushrooms with plant extracts, demonstrated antiproliferative and antimetastatic activity in MDA-MB-231 cells. Oral intake of BreastDefend (100 mg/kg for 4 weeks) exhibited antitumor and antimetastatic actions without damaging any organs in tumor-bearing mice [100]. 

Lanosterol (3β-hydroxy-lanosta-8,24-dien-21-al) and inotodiol [(3β,22R)-lanosta-8,24-diene-3,22-diol] isolated from Chaga mushrooms (*Inonotus obliquus*) suppressed growth in MCF-7 cells [101]. Other reported compounds include trametenolic acid and ergosterol peroxide against MDA-MB-231 cells [102]. Zhang et al. [75] first isolated β-glucan PCM3-II from the mushroom *Poria cocos*, which provokes G1 arrest in a time-dependent manner, as well as downregulation of cyclin D1 and E expressions in MCF-7 cells. Furthermore, the authors also examined reduced viability of MCF-7 cells, the Bax/Bcl-2 ratio, and how apoptosis is downregulating Bcl-2 without affecting Bax [75]. Later, from this mushroom, pachymic acid (PA, a lanostane-type triterpenoid) was isolated, and revealed anticancer effects [103]. In EJ bladder cancer cells, PA induced accumulation of sub-G1 DNA content in a dose-dependent manner [103]. Another study by Ma et al. [104] demonstrated that PA in lung cancer cell lines provokes apoptosis due to activation of the JNK and ER stress pathways. Recently, Jiang and Fan [105] identified PA as the major compound in *Poria cocos* with anticancer property against MDA-MB-231 cells (IC_50_ value, 2.13 ± 0.24 μg/mL), without any cytotoxicity in a normal cell line. 

Gu and Leonard [106] reported anticancer efficacy of 38 mushrooms (edible species) against breast cancer cell lines. Anticancer agents inhibiting tumor growth were identified in the aqueous extracts of *Coprinellus* sp., *Flammulina velutipes*, and *Coprinus comatus*, but never confirmed by clinical studies. *Pleorotus ostreatus* is another mushroom without clinical trials. It inhibited the proliferation of breast and colon cancers via p53-dependent and p53-independent mechanisms. The fungus induced the expression of the tumor suppressor p53 and the cyclin-dependent kinase inhibitor p21 (CIP1/WAF1), but inhibited the phosphorylation of retinoblastoma protein (Rb) in MCF-7 and HT-29 cells, in breast and colon cancer cells, respectively [107]. 

Notwithstanding the promising effects on cancer cell lines and experimental tumors in animals, most of the compounds discussed in this section have yet to be tested in the clinic. This confirms our impression that mushroom compounds may offer considerable perspectives for the development of novel drugs.

## 7. Challenges for Mushroom Constituents as Anticancer Agents

The therapeutic success of polysaccharides, including β-glucans, requires further research into their structure–activity relationships, molecular conformations, receptor-mediated mechanisms, etc. [108,109,110]. As several mushroom species contain β-glucans, the size, molecular weight, structure, solubility, and molecular mechanisms of β-glucan action needs to be taken into consideration [108]. Especially, the role of molecular weight in the pharmaceutical activity of β-glucans needs attention. Indeed, while high-molecular-weight preparations such as scleroglucan are highly efficient, at the same time, low-molecular-weight lentinan has a higher antitumor activity [111,112]. Moreover, individual-specific differential reactivity of β-glucans has been reported in various strains of mice. For example, the anti-β-glucan titer, and increases in the titer by β-glucan administration and the reactivity of peripheral blood leucocytes differs considerably among individuals [108]. 

Solubility in water is another important characteristic of β-glucans, since factors disturbing solubility and pharmaceutical activity of β-glucans are yet to be confirmed. Molecular weight, length, and the number of side chains, the ratios of (1,4), (1,6), and (1,3) linkages, ionization by acid, etc. are discussed by various authors [74,108]. Besides, the mechanism behind intestinal absorption of β-glucans administered orally remains unknown. Various propositions have been made; “nonspecific intestinal absorption, passage of β-glucans through the gap junction in the intestinal epithelium, absorption through intestinal M cells, absorption after binding with Toll-like receptor proteins on the intestinal lumen, and dendritic cell probing” [74,113]. It has been hypothesized that orally administered insoluble β-glucans are later degraded into smaller bioactive oligomers after ingestion [114]. In addition, the differences in structure, solubility, and biological activity of β-glucans derived from plant, yeast, and mushroom sources should be resolved. Although the binding of β-glucans to the dectin-1 receptor (dendritic cell-associated C-type lectin-1) has been demonstrated [115,116], such information for dectin-2 is almost non-existent.

A 43 KDa antitumor protein isolated from *Pholiota nameko* disrupted the mitochondrial transmembrane potential, distorted distribution of cells in distinct cell cycle phases, and demonstrated antiproliferative and apoptosis-inducing activities when tested on MCF7 cells [117]. Later, a novel sterpurane sesquiterpene, known compounds 15-hydroxy-6α,12-epoxy-7βH,10αH,11αH-spiroax-4-ene and 4βH,7βH-hydroxyeremophil-1(10)-en-2-one, were identified from this mushroom and were found to be noncytotoxic at 40 μM when tested against 4 cancer cell lines [118,119]. Isolation and identification of the active compound through bioassay-guided purification requires for further studies. However, triterpenes and aristolane sesquiterpenes, e.g., (24E)-3,4-seco-cucurbita-4,24-diene-3-hydroxy-26,29-dioic acid and (24E)-3,4-seco-cucurbita-4,24-diene-3,26,29-trioic acid, isolated from *Russula lepida*, did not show any cytotoxicity at 50 μM when tested against Huh-7 and EJ-1 cells [120,121].

## 8. Prospects for Development of Drugs from Mushrooms 

Most of the active components such as lentinan, schizophyllan, and krestin, extracted from mushrooms, have high molecular weight compounds. Therapeutic efficacy requires these high-molecular-weight compounds for immunomodulation and other anticancer effects [68,122]. Polysaccharides of high molecular weight cannot be synthesized, while the cost of their production from natural sources, such as through extraction from fruiting bodies, cultured mycelium, or cultured broth, tends to be high. Therefore, more attention should be focused on the development of drugs using low-molecular-weight compound-targeting processes, such as apoptosis, angiogenesis, metastasis, cell cycle regulation, and oncogenic signal transduction cascades [123].

The diversity, easy culture methods, and increasing popularity of mushrooms offer one of the best gifts of nature for new sources of natural products, including pharmaceuticals. This review presents the potential of MM for cancer therapy, and recent development on this subject. Many investigated mushrooms possess interesting direct/complimentary in vitro and in vivo anticarcinogenic effects in mouse models without significant side effects [9]. However, only a few mushrooms and even fewer of their purified molecules have been studied clinically; although, they have already demonstrated inhibition or triggering of specific responses pertinent for cancers, e.g., activating or inhibiting NF-κB, inhibiting proteins, and especially tyrosine kinases, aromatase, and sulfatase, matrix metalloproteinases, cyclooxygenases, DNA topoisomerases and DNA polymerase, inhibiting angiogenesis, and so on. Testing the efficacy of large numbers of low-molecular-weight compounds, individually or in combination with established anticancer treatments, and in suitable dosages, should be an important objective of future clinical studies. 

Currently, information on the anticancer use of MM is rather limited, and the scientific methodological quality of selected clinical studies leaves room for improvement. The existing evidence in many studies only permits preliminary conclusions, although several papers have validated in vitro activity. Interestingly, several in vitro studies on the mechanism of actions have clearly demonstrated immunomodulating effects, namely the proliferation of lymphocytes and alterations in immunoglobulins and cytokines, etc. 

Studies in mouse tumor models have significantly progressed, but clinical studies remain limited. The lack of standardization in preparation methods, large patient sample sizes, modes of administration, and long-term follow-up studies decreases the reliability and validity of those studies [6]. More work needs to be done to justify the role of mushrooms in managing cancer, besides being a part of a healthy diet. There is an urgent need to explore the efficacy and safety of MM in well-planned RCTs, as more and more patients use mushrooms as a co-medication. It is probable that MM could improve QOL during and after conventional cancer therapy.

## 9. Future Prospects

Overall, several clinical reports suggest that mushrooms can control cancerous cell proliferation and may be used as for treatment. In most clinical investigations, mushrooms crude extracts were tested, while few trials used known compounds as anticancer agents [5,6]. In contrast to pure compounds, crude extracts are rarely used in mainstream medicine due to their complexity and unclear mechanisms of action. Thus, bioassay-guided purification of the compounds responsible for the anticancer effects may be useful in yielding potential drug candidates. In addition, more convincing experimental therapeutic evidence and continued efforts are also required to achieve the objectives of potential anticancer drug discovery. Furthermore, advanced artificial intelligence and bioinformatics tools can be used to accelerate target specific anticancer drug discovery [124,125]. 

Several early screening and diagnosis procedures have been used to understand cancer biology, and several instruments and techniques have also been applied to isolate bioactive compounds from natural resources [126,127,128]. However, the complex drug development and validation processes and high failure rates in the translational phase are the most discouraging factors for pharmaceutical companies to pursue natural product-based therapy. Over 90% of drug development candidates are unsuccessful in the clinical translation stage due to several problems, such as drug toxicity, delivery, pharmacokinetics profile, etc. [129]. Thus, strong experimental evidence at the preclinical stage is needed to convene a pharmaceutical company to start clinical development. In this respect, it may be useful to isolate the bioactive compounds, e.g., using bioassay-guided purification. This will help to standardize treatment; although, interactions between multiple bioactive components may prove challenging. 

## 10. Conclusions

Scientific evidence for the use of mushrooms in treating cancer is still limited, and the methodological quality of most of studies could be improved. There are many publications on in vitro and in vivo anticancer properties, but clinical studies are often lacking with proper standardization, inclusion of sufficient numbers of patients, clear preparation methods, sufficient duration of treatment, clear mode of administration, and dosage, etc. Thus, present evidence only allows preliminary conclusions. Moreover, a few studies used combinations of extracts, so that it is unclear which mushroom is responsible for the therapeutic effect. Preparations based on mycelia grown under controlled conditions would probably be more acceptable, but a better understanding is needed of the mechanism of action. As per reports, most mushroom fractions contained polysaccharide-protein conjugate types products and showed more promising antitumor activity. There is convincing (preclinical and clinical) evidence for the immunological effects of mushroom extracts; although, the relationship with the anticancer activity is often not clear. Effects on the immune system may contribute to the improved QOL, and may account for the paucity of anticancer effects in monotherapy; meanwhile, anticancer effects have been observed when mushrooms are combined with other therapeutic modes, whose side effects may also be mitigated. As we learn more about immunotherapy of tumors, the use of mushrooms may find its proper place in the treatment of cancer patients. The immunological effects of mushrooms are typically attributed to polysaccharides, but many mushrooms contain also small molecules secondary metabolites with interesting bioactivities, including for cancer. Pharmaceutical activities of only a few mushrooms have been studied during the past decades; therefore, much remains to be explored. Edible mushrooms especially seem attractive as a source of bioactive compounds, since their safe use in humans has already been established. Moreover, there is a gap in information between Eastern and Western medicine: several mushroom species are used as traditional medicine in Asia, but have barely been studied in Western medicine, perhaps due to the complex nature of the extracts and the absence of acceptable pharmacological purity. In addition, high-quality, long-term, randomized, double-blind, placebo-controlled human clinical studies, which have large sample sizes and are sufficiently powered, using modern statistical and bioinformatics approaches, are needed. Additional studies are desirable to demonstrate which mushroom extracts or compounds are the most effective for specific types of cancer.

## Figures and Tables

**Table 1:** Selected clinical studies of mushrooms with anticancer activity.

Scientific Name	Type of Study	Major Outcomes	Reference
*Agaricus bisporus*	Phase I trial, *n* = 32	Appeared to reduce prostate cancer by decreasing immunosuppressive factors.	[10] *
*Agaricus blazei*	Randomized, placebo-controlled, double-blind clinical trial (RCT), *n* = 40	AndoSan^TM^ as adjuvant therapy to high dose of melphalan improved a few immune-modulating effects. In addition, increase in serum levels (IL-1, IL-5, and IL- 7) and expression of antibodies and killer immunoglobulin receptor (KIR) genes were observed.	[12] *
*Agaricus blazei*	RCT, *n* = 100	Between treated and non-treated groups, there was no significant difference w.r.t. lymphokine-activated killer and monocyte activities among cervical, ovarian, and endometrial cancer patients undergoing chemotherapy. Additionally, several side effects were improved by verum only when treated with mushroom extract	[13] *
*Agaricus sylvaticus*	RCT, *n* = 56	Significant reduction in fasting plasma glucose, total cholesterol, creatinine, aspartate aminotransferase, alanine aminotransferase, IgA, IgM, and systolic and diastolic blood pressure.	[31]
*Agaricus sylvaticus*	RCT, *n* = 46	Improved nutritional status with reduced adverse effects (nausea, vomiting, and anorexia), in patients with breast cancer, stage II and III.	[15]
*Cordyceps sinensis*	Clinical study, *n* = 36	Jinshuibao capsule (containing constituents similar to *Cordyceps sinensis*) restored cellular immunological function, improved quality of life (QOL), but had no substantial effect on humoral immune function.	[32]
*Ganoderma lucidum*	Pilot clinical trial, *n* = 48	Treated breast cancer patients showed significant enhancements in physical well-being and fatigue with a reduced amount of anxiety and depression.	[19]
*Ganoderma lucidum*	Open label, *n* = 36	Ganopoly^®®^ significant increase in mean plasma concentrations of IL-2, IL-6, and IFN-γ, whereas the levels of IL-1 and TNF-α were significantly decreased. The mean absolute number of CD56+ cells was significantly increased, whereas the numbers of CD3+-, CD4+-, and CD8+-expressing cells were just marginally increased compared with baseline levels, with the CD4:CD8 T cell ratios unchanged. PHA responses were enhanced in most patients; and mean NK activity was increased compared with baselines.	[33]
*Ganoderma lucidum*	RCT, *n* = 68	A significant increase in Karnofsky scores compared with placebo among the advanced-stage lung cancer patients. Less disease progression. In addition, several cancer-related symptoms and immune parameters were significantly improved in verum.	[34]
*Ganoderma lucidum*	Controlled clinical Trial, *n* = 198	Decrease in both number and size of colorectal adenomas for the verum group.	[29]
*Grifola frondosa*	Phase I/II, dose escalation trial, *n* = 34	Maitake extracts affects both immunological stimulatory and inhibitory parameters in peripheral blood with treated post-menopausal breast cancer patients.	[20] *
*Lentinula edodes*	Phase II clinical trial, *n* = 74	Mushroom extract failed to reduce by >50% prostate- specific antigen in early stage prostrate cancer patients.	[24] *
*Lentinus edodes*	Clinical trial, *n* = 62	Administration of *L. edodes* extract in prostate cancer patients failed to stabilize or halt progression of disease.	[25] *
*Schizophyllum commune*	Clinical trial, *n* = 220	Tumor-reducing effect in cervical cancer patients with stage II or III. Time to recurrence was longer in in stage II but not stage III cancer, compared with control group; 48-month survival time of patients with stage II but not stage III cancer in the SPG group was significantly longer than in the control group.	[30]
*Trametes versicolor*	Controlled trial, *n* = 60	Significantly improved symptoms of Qi and Yin deficiency in gastric cancer patients after chemotherapy.	[35]

RCT—randomized clinical trial; *—papers also retrieved from SciFinder.

**Table 2:** Summary list of mushroom species studied for anticancer properties.

Cancer Type	In Vitro Study	In Vivo Study	Clinical Trial
Miscellaneous tumors	*Agaricus bisporus, Agaricus blazei, Antrodia camphorata, Grifola frondosa, Phellinus linteus, Phellinus rimosus, Ramaria flava*	*Agaricus blazei, Agaricus sylvaticus, Antrodia camphorata, Amauroderma rude, Cordyceps sinensis, Flammulina velutipes, Ganoderma lucidum, Grifola frondosa, Lentinus edodes, Lepista inversa, Pleurotus nebrodensis, Tricholoma mongolicum*	*Phellinus rimosus*
Bladder	*Phellinus linteus, Poria cocos*	-	-
Blood	*Agaricus blazei, Cordyceps sinensis, Grifola frondosa, Pleurotus ostreatus*	-	*Grifola frondosa*
Breast	*Agaricus bisporus, Agaricus blazei, Amauroderma rude, Antrodia cinnamomea, Antrodia camphorata, Antrodia salmonea, Amauroderma rude, Cordyceps sinensis, Coriolus versicolor, Cortinarius xiphidipus, Fuscoporia torulosa, Ganoderma lucidum, Grifola frondosa, Inonotus obliquus, Laetiporus sulphureus, Lentinus crinitus, Lentinus polychrous, Lignosus rhinocerotis, Lignosus tigris, Marasmius oreades, Phellinus linteus, Phellinus rimosus, Pholiota adiposa, Pholiota nameko, Pleurotus abalones, Pleurotus djamor, Pleurotus highking, Pleurotus nebrodensis, Pleurotus ostreatus, Poria cocos, Tricholoma mongolicum, Xylaria schweinitzii*	*Agaricus bisporus, Agaricus blazei, Amauroderma rude, Antrodia salmonea, Ganoderma lucidum, Lignosus tigris, Phellinus rimosus, Poria cocos, Schizophyllum commune*	*Agaricus bisporus, Agaricus sylvaticus, Coriolus versicolor, Ganoderma lucidum, Grifola frondosa*
Cancer cachexia	-	*Antrodia cinnamomea*	
Cervical	-	-	*Agaricus blazei, Schizophyllum commune*
Chronic hepatitis C infection	-	-	*Agaricus blazei*
Colorectal	*Agaricus bisporus, Agaricus blazei, Antrodia salmonea, Cerrena unicolor, Ganoderma lucidum, Grifola frondosa, Inonotus obliquus, Lentinan, Marasmius oreades, Phellinus linteus, Pleurotus sajor-caju, Pleurotus ostreatus, Pycnoporus sanguineus, Sarcodon aspratus, Taiwanofungus salmoneus*	*Agaricus blazei*	*Agaricus sylvaticus, Ganoderma lucidum, Lentinan*
Endometrial	-	-	*Agaricus blazei*
Gastric	*Agaricus blazei*	-	*Trametes versicolor, Lentinan*
Liver	*Agaricus blazei, Auricularia auricula-judae, Cordyceps sinensis, Coriolus versicolo, Lentinan, Russula alatoreticula, Thelephora aurantiotincta, Tricholoma mongolicum, Xylaria schweinitzii*	*Agaricus blazei, Auricularia auricula-judae, Ganoderma lucidum, Phellinus linteus, Schizophyllum commune*	*Coriolus versicolo, Lentinan*
Lung	*Agaricus blazei, Antrodia cinnamomea, Cordyceps sinensis, Flammulina velutipes, Ganoderma lucidum, Grifola frondosa, Inonotus obliquus, Lentinula edodes, Phellinus linteus, Lentinus squarrosulus, Pleurotus nebrodensis, Pleurotus nebrodensis*	*Poria cocos*	*Ganoderma lucidum, Grifola frondosa*
Lymphoma in dogs	-	-	*Grifola frondosa*
Myeloma	-	-	*Agaricus blazei*
Nasopharyngeal	-	-	*Ganoderma lucidum*
Ovarian	*Antrodia salmonea*	-	*Agaricus blazei, Agaricus bisporus, Volvariella volvacea*
Pancreatic	*Agaricus blazei*	-	-
Prostate	*Fuscoporia torulosa, Ganoderma lucidum, Lentinula edodes, Phellinus linteus*	-	*Agaricus bisporus, Lentinula edodes*
Testicular	*Cordyceps sinensis*	-	-
Other advanced cancers	-	-	*Antrodia cinnamomea, Cordyceps sinensis, Ganoderma lucidum, Lentinula edodes*

“-”—no data available.

**Table 3:** Scores for each species of mushrooms regarding its anticancer properties.

Name of the Mushroom	Type of Cancer	Type of Studies (References)					Overall Strength of Recommendation
		In Vitro	In Vivo	In Silico	Clinical Study	Active Constituents	
*Agaricus bisporus*	Breast, colon, prostate cancer	***	**	***	**	**	**
*Agaricus blazei*	Several types of cancer: myeloma, leukemia, chronic hepatitis C infection, breast, cervical, ovarian, lung, pancreatic, and endometrial	***	***	-	***	***	***
*Agaricus sylvaticus*	Colorectal and breast cancer	***	**	-	***	*	**
*Amauroderma rude*	Breast cancer	***	*	-	-	**	*
*Antrodia cinnamomea*	Breast and lung cancer	***	***	-	*	**	**
*Antrodia camphorata*	Miscellaneous tumor	**	*	-	-	*	*
*Antrodia salmonea*	Breast, colon, and ovarian cancer	***	**	-	-	*	*
*Auricularia auricula-judae*	Hepatoma	*	-	*	-	*	*
*Cerrena unicolor*	Colon cancer, miscellaneous tumors	***	*	-	-	-	*
*Cordyceps sinensis*	Lung and testicular cancer	***	*	**	*	**	**
*Coriolus versicolor*	Breast, gastric, and liver cancer	***	***	***	***	**	***
*Cortinarius xiphidipus*	Several types	*	-	-	-	-	-
*Flammulina velutipes*	Lung cancer and miscellaneous tumor	**	-	*	-	***	*
*Fuscoporia torulosa*	Brest and prostate cancer	*	-	-	-	-	-
*Ganoderma lucidum*	Breast, lung, colorectal, and Nasopharyngeal cancer	***	***	***	***	***	***
*Grifola frondosa*	Blood, breast, and lung cancer	***	**	*	***	***	***
*Inonotus obliquus*	Breast cancer	***	**	*	-	***	**
*Lentinus edodes*	Breast, lung, colorectal, gastric, and liver cancer	***	**	**	***	***	***
*Lentinus squarrosulus*	Lung cancer	*	-	-	-	-	-
*Lepista inversa*	Several cancer cell lines	*	-	-	-	-	-
*Lignosus rhinocerotis*	Breast cancer	**	*	*	-	**	*
*Lignosus tigris*	Breast cancer	**	*	-	-	*	*
*Marasmius oreades*	Colon and breast cancer	**	*	-	-	*	*
*Phellinus linteus*	Colon, liver, lungs, and prostate cancer	***	**	*	-	***	**
*Phellinus rimosus*	Colon and liver cancer	***	*	-	-	**	*
*Pholiota nameko*	Breast cancer	**	*	-	-	*	*
*Pleurotus abalones*	Breast cancer	**	*	-	-	*	*
*Pleurotus highking*	Breast cancer	**	*	-	-	*	*
*Pleurotus nebrodensis*	Liver, lungs, and breast cancer	***	*	-	-	**	*
*Pleorotus ostreatus*	Blood, lungs, and breast cancer	***	**	*	-	*	*
*Poria cocos*	Breast and pancreatic cancer	***	**	**	*	***	**
*Pycnoporus sanguineus*	Colon cancer	*	-	-	-	-	-
*Ramaria flava*	Liver cancer	**	-	-	-	-	-
*Russula alatoreticula*	Liver cancer	*	-	-	-	-	-
*Schizophyllum commune*	Breast, liver, and cervical cancer	***	*	-	*	**	**
*Thelephora aurantiotincta*	Liver cancer	*	-	-	-	-	-
*Taiwanofungus salmoneus*	Colon and liver cancer	***	*	-	-	**	*
*Tricholoma mongolicum*	Breast and liver cancer	***	-	-	-	*	*
*Xylaria schweinitzii*	Breast, liver, and lung cancer	*	-	-	-	*	-

***—excellent, >5 studies; **—good, 3–5 studies; *—poor, 1–2 studies; “-”—no study.

## Data Availability

Data is contained within the article.

## Supplementary Materials

The following are available online at https://www.mdpi.com/article/10.3390/ph15020176/s1, Table S1: Clinical studies of mushrooms with anticancer activity (source https://pubmed.ncbi.nlm.nih.gov/), Table S2: Other trials of mushroom(s)/compound(s) with anticancer activity not retrieved from PubMed (source https://scholar.google.com/), Table S3: Clinical studies of mushroom form other databases (https://www.clinicaltrials.gov/), Table S4: Characteristics of studies related to anticancer property of mushroom(s), database source (https://pubmed.ncbi.nlm.nih.gov/), Table S5: Summary of active constituents of mushroom(s) and their mechanism of action.

## Abbreviations

AE	adverse event
AHCC^®®^	active hexose correlated compound
FFLZ	fucose-containing fraction of *G. lucidum*
HCC	hepatocellular carcinoma
HP	hematologic parameters
IC50	half-maximal inhibitory concentration
LOA	loss of appetite
MDS	myelodys plastic syndromes
MM	medicinal mushrooms
OS	overall survival
QOL	quality of life
RCT	randomized, placebo-controlled, double-blind clinical trial
ROS	reactive oxygen species
SDL	superfine dispersed lentinan
SPG	polysaccharide schizophyllan
