# Supplementary material for: Anticancer Activities of Mushrooms: A Neglected Source for Drug Discovery
Source: Pharmaceuticals (Basel). 2022 Jan 31;15(2):176. doi: 10.3390/ph15020176 (PMC8876642; doi:10.3390/ph15020176)
Supplement: Supplementary file 1 [file pharmaceuticals-15-00176-s001.zip › pharmaceuticals-1562790-supplementary.pdf]

Review

Anticancer activities of mushrooms: a neglected source for drug discovery

Sujogya Kumar Panda[1,2,3*], Gunanidhi Sahoo[2,] Shasank S. Swain[4] and Walter Luyten[3]

[1] Center of Environment, Climate Change and Public Health, RUSA 2.0, Utkal University, Bhubaneswar-751004, India. Email- sujogyapanda@utkaluniversity.ac.in (SKP)
[2] Department of Zoology, Utkal University, Bhubaneswar-751004, India. Email-sahoogunanidhi.zool@utkaluniversity.ac.in (GS)
[3] Department of Biology, KU Leuven, 3000 Leuven, Belgium. Email-walter.luyten@kuleuven.be (WL)
[4] Division of Microbiology and NCDs, ICMR-Regional Medical Research Centre, Bhubaneswar 751023, India. Email-swain.shasanksekhar86@gmail.com (SSS)
* Correspondence: Email-sujogyapanda@utkaluniversity.ac.in (SKP)

Supplementary materials

Table S1. Clinical studies of mushrooms with anticancer activity (source https://pubmed.ncbi.nlm.nih.gov/).

Scientific name [English name]	Study design					Type of cancer/ Stage	Outcomes	Reference
	Type of study	Number of patients	Parameters	Dose	Duration of treatment			
Agaricus bisporus (J.E. Lange) Imbach (1946); *Synonym Psalliota hortensis* f. *bispora* J.E. Lange (1926); [white button mushroom]	Phase I trial	32	Determination of the effect on biochemical parameters blood, serum and evaluation of toxicity.	Mushroom powder: 6 dosages start with 4 g/d. The highest dosage was capped at 14 g/d (i.e., 28 tablets daily).	-	Prostate cancer	Appeared to reduce prostate cancer by decreasing immune-suppressive factors.	[1] *
Agaricus blazei auct. non Murrill (1945); synonyms *Agaricus subrufescens* Peck (1893); *Agaricus rufotegulis* Nauta (1999); *Agaricus blazei* Murill Kyowa (AbMK)	Randomized, placebo-controlled, double-blind clinical trial (RCT)	100	Immunological status, side effects, quality of life (QOL).	Daily oral consumption of mushroom extracts for 3 weeks.	Every three weeks, at least for 3 cycles after treatment of chemo-therapy.	Cervical, ovarian, endometrial cancer undergoing chemo-therapy.	Between treated and non-treated groups, there was no significant difference w.r.t. lymphokine-activated killer and monocyte activities. Also, several side effects were improved by *verum* only when treated with mushroom extract.	[2] *
Agaricus blazei	Phase I clinical trial	5	Effect on gene expression in peripheral blood cells.	β-glucan extract.	-	Chronic hepatitis C infection.	No changes in mRNA level (G-protein coupled receptor signaling pathway, cell cycling, and	[3] *

							transcriptional regulation).	
Agaricus blazei	RCT	40	Immuno-modulatory effects (changes in serum levels and hematological parameters (HP), involvement of genes in immune activation.	AndoSan™ (*Agaricus blazei*=82.4%, *Hericium erinaceus*=14.7% and G*rifola frondose*= 2.9%); 60 mL/d.	7 weeks	Multiple myeloma undergoing high dose chemo-therapy, phase II.	AndoSan™ as adjuvant therapy to high dose of melphalan improved a few immune-modulating effects. In addition, increase in serum levels (IL-1, IL-5 and IL- 7) as well as expression of antibodies and killer immunoglobulin receptor (KIR) genes.	[4] *
Agaricus sylvaticus Schaeff. (1774); [scaly wood / blushing wood / pinewood mushroom]	RCT	56	QOL indicators.	Extract (30 mg/kg/ d; 6 tablets per day).	6 months	Colorectal cancer, stage I-III	QOL improved after post-surgical phase with dietary supplement containing *A. sylvaticus*.	[5]
Agaricus sylvaticus	RCT	56	Complete hemogram, serum iron, and fasting glycaemia.	Extract (30 mg/kg/ d; 6 tablets per day).	6 months	Colorectal cancer, stage I-III	Significant increases in hemoglobin, hematocrit, erythrocytes and neutrophil levels: platelet count significantly reduced but remained within normal levels. Reduced glycaemia levels.	[6]

Agaricus sylvaticus	RCT	56	Glucose, total cholesterol, creatinine, alkaline phosphatase, bilirubin, aspartate aminotransferase and alanine aminotransferase; IgA, IgG and IgM; total proteins and protein fractions; blood pressure.	Extract (30 mg/kg/d; 6 tablets per day).	6 months	Colorectal cancer, stage I-III	Significant reduction of fasting plasma glucose, total cholesterol, creatinine, aspartate aminotransferase, alanine aminotransferase, IgA, IgM, systolic and diastolic blood pressure.	[7]
Agaricus sylvaticus	RCT	56	Three fasting glycaemia evaluations at start of treatment and after 3 and 6 months.	Extract (30 mg/kg/d; 6 tablets per day).	6 months	Colorectal cancer, stage I-III	Significantly reduced fasting glycemia level after 6 months in post-surgery colorectal cancer patients.	[8]
Agaricus sylvaticus supplementation	RCT	46	Chemotherapy-associated adverse effects.	Dietary supplementation containing mushroom (2.1 g/d) or placebo (6 tablets per day).	6 months	Breast cancer, stage II and III.	Improved nutritional status with reduced adverse effects (nausea, vomiting and anorexia).	[9]
Antrodia cinnamomea Chang & Chou (1995) [AC mushroom]	RCT	37	6-month overall survival (OS), disease control rate, QOL, adverse event (AE).	20-mL oral formulations prepared from *A. cinnamomea* twice a day.	30 days	Advanced cancer.	*A. cinnamomea* in combination with chemotherapy was not effective to improve outcome in cancer patients.	[10] *

							"The combination may present a potential risk of lowered platelet counts. Adequately powered clinical trials will be necessary to address this question.".	
Coriolus versicolor (L.) Quél. (1886); synonym *Trametes versicolor* (L.) Lloyd (1920) [Turkey tail]	RCT	15	QOL, progression-free survival, OS and toxicity.	Standard continuous daily dose of 2.4 g. Median treatment duration was 1.5 cycles (5.9 weeks) and 3 cycles (12.1 weeks) for placebo and mushroom, respectively.	-	Hepatocellular carcinoma (HCC)	HCC patients taking mushroom had better social and emotional functioning scores, as well as less pain and loss of appetite (LOA) compared to placebo-treated subjects.	[11] *
Trametes versicolor	Phase I, two-center, dose escalation study	11	Immune parameters	Chemo- and radiotherapy-treated women were given freeze-dried mycelial powder doses 3, 6 and 9g/d.	6 weeks	Breast cancer	Increase in lymphocyte counts and NK cell functional activity with dose 6 and 9 g/d. In addition, increase in CD8(+) T cells and CD19(+) B cells, but not CD4(+) T cells.	[12]
Ganoderma lucidum	Pilot clinical trial	48	FACT-F, HADS, and EORTC QLQ-	Spore powder of *Ganoderma lucidum*	Four weeks.	Patients with reast cancer, stage I-III with	Treated cancer patients showed significant	[13]

			C30 questionnaires	1 g three times a day for 4 weeks		cancer-related fatigue undergoing endocrine therapy	enhancements in physical well-being and fatigue with a reduced amount of anxiety and depression.	
Grifola frondosa (Dicks.: Fr.) Gray	Prospective, non-controlled, clinical trial	15	Blood and chemistry profile, body weight, and tumor measurements.	Mushroom extract (dose of 3 drops/kg/d). Doses divided into 2 and given 1 hour before feeding.	12 weeks	Lymphoma in dogs	Administration of maitake extract did not improve significantly lymph node size by greater than 50% (objective response) in any dog	[14] *
Grifola frondosa	Phase I/II, dose escalation trial	34	Safety and tolerability	Liquid polysaccharide extracts (oral administration of 0.1, 0.5, 1.5, 3.0, or 5 mg/kg twice daily for 3 weeks.	3 weeks	Post-menopausal breast cancer patients, free of disease after initial treatment.	Maitake extracts affects both immunological stimulatory and inhibitory parameters in peripheral blood.	[15] *
Grifola frondosa	Phase II study	21	Innate immune function in myelodys-plastic syndromes (MDS)	Powder (3 mg/kg), oral administration of twice per day.	12 weeks	Blood cancer.	Maitake has beneficial immunomodulatory potential in MDS.	[16] *
Lentinula edodes (Berk.) Pegler (1976), Synonym: *Lentinus edodes* [Shiitake]	Phase II clinical trial	74	Adverse effects, change of prostate specific antigen and QOL.	Mushroom extract, active hexose correlated compound (AHCC®, 4.5 g/d), an extract prepared from	6 months	Prostate cancer, early stage	Mushroom extract failed to reduce by >50% prostate-specific antigen.	[17] *

				cultured mycelia of *Lentinula edodes.*				
Lentinula edodes	Exploratory clinical trial	7	QOL score, lymphocyte activity and serum immune indices.	*Lentinula edodes* mycelia extract	-	Chemotherapy for breast or gastrointestinal cancer.	The concomitant use of *Lentinula edodes* mycelia extract with chemotherapy is safe and improves the QOL score, NK cell activity and immunosuppressive acidic protein levels in cancer patients.	[18]
Lentinus edodes	Clinical trial	62	Prostate-specific antigen level.	Oral administration of capsules thrice daily.	6 months	Prostate cancer	Administration of *L. edodes* extract in prostate cancer patients failed to stabilize or halt progression of disease.	[19]*
Lentinus edodes, Shiitake mushroom product, (AHCC®)	Clinical trial Evaluation of safety and effectiveness of AHCC for adverse effects and QOL in patients with cancer.	24	Blood test, EORTC QLQ-C30 questionnaire, and DNA levels of herpes virus type 6 (HHV-6) in saliva.	Intake of AHCC® p.o., 3 g/d.	.	Various cancer, treated with chemotherapy.	AHCC® improved the QOL scores, without hematotoxicity and hepatotoxicity and decreased the levels of herpesvirus in the saliva of patients who received chemotherapy.	[20]*

(-) Data not available; *Papers also retrieved from SciFinder

Table S2. Other trials of mushroom(s)/compound(s) with anticancer activity not retrieved from PubMed (source https://scholar.google.com/).

Scientific name [English name]	Study design					Type of cancer/ Stage	Outcomes	Reference
	Type of study	Number of patients	Parameters	Dose	Duration of treatment			
Cordyceps sinensis	Clinical study	36	Immune functions	-	-	Several with advanced stage	Jinshuibao capsule (containing constituents similar to *Cordyceps sinensis*) restored cellular immunological function, improved QOL, but had no substantial effect on humoral immune function.	[21]
Ganoderma lucidum	Open label	36	Immune functions: mean mitogenic reactivity to phytohemagglutinin, mean counts of CD3, CD4, CD8, and CD56, mean plasma concentrations of interleukin (IL)-2, IL-6, and interferon (IFN)-γ, or NK activity.	5.4 g/d Ganopoly® (*Ganoderma lucidum* polysaccharides from Encore International Corp., Auckland, New Zealand).	12 weeks.	Lung cancer, advanced stage	Ganopoly did not significantly alter the tested immune parameters.	[22]
Ganoderma lucidum	Open label	36	Immune parameters (cytokines, T cell subsets, mitotic response to phytohaemagglutinin and natural killer (NK) activity).	1800 mg/d Ganopoly®.	12 weeks	Advanced stage cancer.	Ganopoly® significant increased mean plasma concentrations of IL-2, IL-6, and IFN-γ, whereas the levels of IL-1 and TNF-α were significantly decreased. The mean absolute number of CD56+ cells was significantly increased, whereas the numbers of CD3+, CD4+, and CD8+ were just marginally increased compared to baseline levels, with the CD4:CD8 T cell ratios unchanged. PHA responses were	[23]

							enhanced in most patients; and mean NK activity was increased compared to baselines.	
Ganoderma lucidum	RCT	68	Karnofsky performance status, immunological parameters.	Extract, 600 mg thrice daily.	12 weeks	Lung cancer, advanced stage.	A significant increase in Karnofsky scores compared to placebo. Less disease progression. In addition, several cancer-related symptoms and immune parameters were significantly improved in *verum*.	[24]
Ganoderma lucidum	RCT	72	HP such as hemoglobin %, RBC, WBC, conversion rate of lymphocytes, NK cell levels etc.	Oral administration of Wuse - Lingzhi – Jiaonang formulation during radiotherapy	-	Nasopharyngeal cancer	Administration of this mushroom along with radiotherapy could not reduce the side effects but in some extents improved a few immune function parameters.	[25]
Ganoderma lucidum	Controlled Clinical Trial	198	Size, site and macroscopic type of all adenomas.	Extract, 1.5 g/d.	12 months	Colorectal adenoma	Decrease in both number and size of adenomas for the verum group.	[26]
Lentinan	A multi-institutional randomized prospective protocol	-	QOL	Patients administered lentinan, tegafur and cisplatin.	-	Gastric cancer, advanced stage	Lentinan could prolong survival and improved QOL of gastric cancer patients.	[27]
Lentinan	A multi-center clinical study	80	QOL	-	-	Colorectal cancer, advanced stage	A significant improvement in QOL scores after 12 weeks of treatment with lentinan.	[28]
Lentinan	Randomized controlled trial	78 (136 tumors)	Parameters like survival duration, tumor necrosis and recurrence rate etc.	500 mg/d.	18 months	HCC	Administration of lentinan along with other therapy was advantageous to increase survival time as well as decrease chances of recurrence rate.	[29]

Lentinan	A multi-center study	36 (out of 40 patients)	Rate of lentinan-binding cells in CD14+ monocytes.	Supplementary food containing superfine dispersed lentinan (SDL).	7 to 12 weeks	HCC	Effective for survival of HCC patients.	[30]
Schizophyllum commune Fr.: Fr.	Clinical trial	220	Tumor responses, time of recurrence survival, immunologic parameters and side effects.	Polysaccharide Schizophyllan (SPG): intramuscularly once or twice weekly (40 mg).	Administration as long as possible till 18 months.	Cervical cancer, stage II or stage III who had been given irradiation, concomitantly	Tumor-reducing effect in patients for either stage. Time to recurrence was longer in in stage II but not stage III cancer, compared with control group. At 48-months survival time of patients with stage II but not stage III cancer in the SPG group was significantly longer than in the control group. groups.	[31]
Trametes versicolor	Controlled trial	60	-	Orally administered Yunzhi glycopeptide, while the control group was treated with squalanol.	-	Gastric cancer	Significant improved symptoms of Qi and Yin deficiency in patients after chemotherapy.	[32]

(-) Data not available

Table S3. Clinical studies of mushroom form other databases (https://www.clinicaltrials.gov/).

Scientific name	Study design				Type of cancer/ Stage	Outcome	Identifier no.
	Type of study	Number of patients	Dose	Duration of treatment			
Agaricus bisporus	A randomized, phase II trial	132	Variable dose.	12 to 48 weeks	Prostate cancer (multiple stages)	This study aims to determine if a white button supplement can reduce prostate-specific antigen. Not yet recruiting.	NCT04519879
Agaricus bisporus	interventional clinical trial	16	Variable dose.	12 weeks	Breast cancer	To determine the efficacy of white button, mushroom postmenopausal breast cancer	NCT00709020

						survivors. Study completed but no results posted.	
Coriolus versicolor	RCT pilot clinical study, phase IV	60	3.5 g/d.	6 months	Breast cancer	To study the effects of Yunzhi extract as adjuvant in the treatment process. Study completed but no results posted.	NCT00647075
Grifola frondosa	A randomized, interventional clinical trial	480	Two tablets, twice a day.	42 days	Lung neoplasms and breast carcinoma	To study the efficacy to treat lung neoplasms and breast carcinoma. Study completed but no results posted.	NCT02603016
Trametes versicolor	Clinical trial, phase I	11	Capsules, twice a day (3 g/d for first week), subsequently increasing dose every week up to 24 g/d.	-	Breast cancer	To determine the maximum tolerated dose of the mushroom extract in women with stage I-III breast cancer, who have recently completed standard post-surgery radiotherapy. Study completed but no results posted.	NCT02568787

Table S4: Characteristics of studies related to anticancer property of mushroom(s), database source (https://pubmed.ncbi.nlm.nih.gov/)

Scientific name [English name]	Extract/fraction/ compound	Type of cancer	*In vitro* cell line/ *In vivo*	Dose	Mechanism	Reference
Agaricus bisporus	Ethyl acetate fractions. Mostly encompass unsaturated fatty acids viz. linoleic acid, linolenic acid and conjugated linoleic acid	Breast cancer	MCF-7aro (in vitro); female BALB/c *nu-nu*	100 mg/L. 100 µL of a 4× concentrated extract (in water)	Inhibited testosterone-induced cell proliferation	[33]
Agaricus bisporus	ABL	Colon cancer, breast cancer	HT29, MCF-7, Caco-2, Rama-27 rat mammary fibroblasts	25 µg/mL	inhibits incorporation of $[^3H]$-thymidine into DNA	[34]
Agaricus bisporus	Lectin-like mannose-binding protein (orf239342)	Breast cancer	MCF-7	-	Exerted mannose inhibitable antiproliferative activity	[35]
Agaricus bisporus	Lectin	Changes in cell cycle and related signaling pathways	In vitro, hRPE1 and hRPE3	20 or 90 µg/mL for 3 days	Extract hypophosphorylated Akt, and as a result attenuates cell proliferation.	[36]
Agaricus blazei	Acidic RNA protein complex, FA-2-b-β	Chronic myeloid leukemia	CML K562 cells	-	Anti-proliferative potency as well as pro-apoptotic effects.	[37]
Agaricus blazei	Agarol, an ergosterol	Several type of cancer e.g. lung, gastric, tongue	A549, MKN45, HSC-3, and HSC-4 human	-	Agarol induces caspase-independent apoptosis through a mitochondrial pathway.	[38]

			carcinoma cell lines			
Agaricus blazei	Polysaccharide *Agaricus blazei* Murill (pAbM)	Several tumors	Toll-like receptor 2-deficient (TLR2$^{-/-}$) and Wild-type C57BL/6 mice	2 mg per mouse; equivalent to 100 mg/kg, for 4 weeks or control PBS (*n* = 5 per group).	Anti-tumor effect of pAbM was dependent on Gr-1(+) CD11b(+) monocytes, but neither on CD8(+) T cells nor CD4(+) T cells.	[39]
Agaricus blazei	beta-(1-6)-D: -glucan	Lung cancer	Lewis lung cancer 3LL cells	-	β-glucan decreases pulmonary metastasis of 3LL cells	[40]
Agaricus blazei	Agaritine, a hydrazine-containing compound	Leukemia cancer	U937 cells	10µg/mL of agaritine for 48h	Agaritine induces apoptosis via caspase activation through cytochrome c release from mitochondria.	[41]
Agaricus blazei	Polysaccharides, pAbM	Pancreatic cancer	RAW 264.7	500, 1000, and 2000 µg/mL	pAbM regulates cell-mediated immunity	[42]
Agaricus blazei	Polysaccharide-protein complex	Several tumors	Sarcoma 180 in mice, as well as other carcinomas	10 and 20 mg/kg/d x 10	Macrophage activation and alterations of the C3 are essential for the induction of an antitumor effect.	[43]
Agaricus blazei	Polysaccharide, LMPAB	Hepatic cancer	*In vivo* BALB/c mice; *in vitro* hepatic cancer cells, BEL-7402	-	In the *in vivo* model, the mRNA and protein levels of vascular endothelial growth factor level in tumor tissues were down-regulated.	[44]
Agaricus blazei	A polysaccharide, α-(1-->4)-glucan-β-(1-->6)-glucan-protein complex	Tumor	Sarcoma 180 mice, tumor growth	-	The glucan-protein complex polysaccharide showed significant antitumor effects.	[45]

Agaricus blazei	β-glucan	Hepatic cancer	HepG2 cells	50 µg/mL β-glucan	β-glucan significantly suppressed the expression of the *ERCC5* gene. But no significant change was observed in the CASP9 transcript level.	[46]
Agaricus blazei	*A. blazei* water extract with marine phospholipids	Colon cancer	*In vivo*, sp2 tumor bearing BALB/c nu/nu mice (male); *Iin vitro* Caco-2 cells	0.5 mg/mL (total administration 105 mg/mouse)	*A. blazei* water extract with marine phospholipids is beneficial in myeloma sp2 therapy without any side effect.	[47]
Agaricus blazei	Sodium pyroglutamate	Lewis lung carcinoma (LLC)	Direct inhibition of angiogenesis induced by solid tumors	-	Both antitumor and antimetastatic action may be linked to a decrease of immune response caused by the tumor growth and tumor-induced neovascularization.	[48]
Agaricus sylvaticus	-	Walker 256 tumor	80 rats, divided into 4 groups.	Sacrifying was done after 12 days of treatment	In Walker 256 tumor rats, extract is able to decrease anemia as well as significant progress on several biochemical parameters.	[49]
Amauroderma rude	-	Breast cancer	*In vitro, in vivo*	Inhibit cancer cell survival and induce apoptosis	Effects of extract found with extensive cell death, (decreased proliferation rate, as indicated by Ki67 staining) as well as increased apoptosis (as indicated by TUNEL	[50]

					staining), which may be due to suppression of c-myc expression.	
Antrodia cinnamomea Chang & Chou (1995) [AC mushroom]	-	Breast cancer	*In vitro*	20-80 μg/mL	AC mushroom extract suppressed the phosphorylation of ERK1/2, p38, and JNK1/2 as well as directed to a dose-dependent inhibition on NF-κB binding and activation.	[51]
Antrodia cinnamomea	-	Breast cancer	HER-2/*neu*-overexpressing MDA-MB-453 and BT-474 cells	-	Apoptotic cell death (associated with sub-G1 accumulation), DNA fragmentation, mitochondrial dysfunction, cytochrome *c* release, caspase-3/-9 activation, PARP degradation, and Bcl-2/Bax dysregulation.	[52]
Antrodia cinnamomea	Cordycepin and zhankuic acid A	Lung cancer	Gelatin zymography assay, human lung adenocarcinoma CL1-0	-.	Anti-migration action in human adenocarcinoma CL1-0 cells through the MAPK and PI3K/AKT signalling pathways.	[53]
Antrodia cinnamomea	-	Cancer cachexia	*In vivo* muscle atrophy, mice (lung tumor-) treated with chemotherapy	-	Extract has a protective effect due to attenuating muscle proteolysis, pro-inflammatory cytokine production, and anorexia, and activating IGF-1-	[54]

					dependent protein synthesis.	
Antrodia cinnamomea	-	Breast cancer	Apoptosis, in vitro, MCF-7 as well as tamoxifen-resistant MCF-7	-	Extract induces apoptosis and "suppresses the mRNA expression of *skp2* (S-phase kinase-associated protein 2) by increasing the expressions of miR-21-5p, miR-26-5p, and miR-30-5p in MCF-7 and tamoxifen-resistant MCF-7 cells".	[55]
Antrodia camphorata	dehydroeburicoic acid	Anti-tumor	HL 60 cells xenograft animal model	-	Reduction of tumor size and weight without hampering mouse body weight	[56]
Antrodia salmonea	-	Breast tumor	MDA-MB-231-luciferase-injected nude mice	-	"Both mitochondrial (caspase-9/caspase-3/PARP) and death-receptor (caspase-8/FasL/Fas) signaling pathways are involved in execution of apoptosis".	[57]
Antrodia salmonea	-	Breast tumor	*In vitro* (MDA-MB-231), *in vivo* (an athymic nude mouse model).	-	Administration of this mushroom, was effective by delaying the incidence of the tumor as well as reducing the growth.	[58]
Antrodia salmonea	-	Breast tumor	*In vitro* (MDA-MB-231), *in vivo* (BALB/c-nu mice), several immunofluo-	-	Histological analysis established that treatment with *A. salmonea* can reduce tumor metastasis and upregulated E-	[59]

			rescence assays, Western blotting, RT-PCR, luciferase activity and wound invasion		cadherin expression in biopsied lung tissues.	
Antrodia salmonea	-	Ovarian cancer	*In vitro* (SKOV-3 or A2780)	-	Treated extract-induced apoptosis "associated with the suppression of human epidermal growth factor receptor-2 (HER-2/neu) and phosphoinositide 3-kinase (PI3K)-protein kinase B (AKT) expression in HER-2/neu overexpressing SKOV-3 cells".	[60]
Antrodia salmonea	-	Colon cancer	*In vitro* (SW620, HCT116, and HT29)	-	Treated extract induced cytoprotective autophagy and apoptosis through extracellular signal-regulated kinase (ERK) signaling cascades.	[61]
Amauroderma rude	Polysaccharide F212	Tumour growth	*In vivo*, lymphocyte proliferation		Increased macrophage metabolism, lymphocyte proliferation, and antibody production	[62]
Amauroderma rude	Ergosterol	Breast cancer	Murine cancer cell line B16, MDA-MB-231	Different doses	"Ergosterol-mediated suppression of breast cancer cell viability occurred through apoptosis and that	[63]

					ergosterol up-regulated expression of the tumor suppressor Foxo3”	
Auricularia auricula-judae	β-(1 → 3)-d-glucan	Hepatoma cancer	H22 tumor growth	5 mg kg^{-1}	The water-soluble glucan triggered cell apoptosis, shown by the activation of caspase 3/9 and down-regulated tumor angiogenesis factors VEGF and CD31.	[64]
Cerrena unicolor	-	Colon cancer	HT-29 and epithelial cells CCD 841 CoTr	Concentration range of 25-200 µg/mL	Extracts induced programmed cell death in HT-29 while less in CCD 841 CoTr, without substantially lowering levels of necrosis in both cell lines.	[65]
Cordyceps sinensis	Ethyl acetate extract ergosterol (major constituent) and trace amount of cordycepin, adenosine and carbohydrates.	Several cancer type	*In vitro*; MCF-7, HL-60 human promyelocytic leukemia, HepG2 and B16 melanoma in C57BL/6 mice.	0.05 g/kg-d	Mycelium has strong anti-tumor activity.	[66]
Coriolus versicolor (L.) Quél. (1886); synonym *Trametes versicolor* (L.) Lloyd (1920) [Turkey tail]	-	Breast cancer	T-47D, MCF-7, MDA-MB-231, BT-20	-	Extract has a strong dose-dependent effect by suppressing proliferation of the three breast tumor cell lines, possibly via apoptosis induction, differentially dependent	[67]

					on p53 and Bcl-2 expression.	
Coriolus versicolor	-	Breast cancer	Mouse mammary 4T1 carcinoma	-	Extracts found to be effective as anti-tumor, anti-metastasis and immunomodulation agents, and could protect bone destruction.	[68]
Cortinarius xiphidipus, fruiting body of ectomycorrhizal fungi	Ten sterols were identified, most potent is ergosta-4, 6, 8(14), 22-tetraen-3-one	Several cancer type	*In vitro*: Neuro-2a, Saos-2, MCF7 and LNCaP-C42	-	Sterols are potential source of anticancer agents.	[69]
Flammulina velutipes (W.Curt.: Fr.) Singer	Proflamin (PRF), an acidic glycoprotein with molecular weight 13 ± 4 kDa	Tumour	Immunomodulation and antitumor	Oral administration to mice (doses, 1–30 mg/kg)	Post-surgery treatment with a specific fraction EA6 can inhibit significantly the growth of tumor, mediated by $CD4^+$ T cells.	[70]
Flammulina velutipes	FIP-fve is a protein	Lund cancer	*In vitro*, cell proliferation of A549	-	FIP-fve inhibits lung cancer cell migration via RacGAP1 and increase p53 expression, and downstream gene p21, on Western blot	[71]
Fuscoporia torulosa	One novel steroid and 10 known compounds including oleanonic acid	Brest and prostate cancer	*In vitro*, MCF-7 PC-3, Acetylcholinesterase (AChE) and butrylcholinesterase (BChE)	-	Oleanonic acid, exhibited cytotoxic effects against MCF-7 and potent BChE inhibitory activity.	[72]

Ganoderma lucidum	Polysaccharides	Brain tumors	Glioma-bearing rats		Significant improvements were observed with parameters such as serum interleukin-2, TNF-α, INF-γ, as well as enhanced cytotoxic activity of NK and T cells, promoting the functional maturation of dendritic cells. This leads to prolonged survival of rats by inhibition of glioma growth.	[73]
Ganoderma lucidum	Triterpenes	Prostate cancer	Cell viability, migration, invasion and apoptosis in DU-145	Dose, 2 mg/mL	Extract inhibits cell viability in a dose- and time-dependent manner by the regulation of matrix metalloproteases, as well as induced apoptosis of DU-145 cells.	[74]
[*Ganoderma lucidum* (Leyss. Ex Fr.) Karst.] and [*Ganoderma sinense* Zhao, Xu et Zhang]	12 active compounds, several known ones: ganoderic acid, kaemferol, genist ein and ergostero l).	Hepatocellular sarcoma	*In vivo* and *in silico* study	Hepa1-6-bearing C57 BL/6 mice	Four hub target genes, (NR3C2, AR, ESR1 and PGR), might act as potential treatment markers in tumor.	[75]
Grifola frondosa	D-fraction	Breast cancer	*In vitro*, MCF7	Variable doses, 18-367 mg/mL	D-fraction has pro-apoptotic activity, reducing cancer cell viability.	[76]
Inonotus obliquus	Eight triterpenoids	Lung cancer	Cell viability and apoptosis	-	Three compounds: β-hydroxylanosta-8,24-dien-	[77]

	including a novel one: 3β-hydroxy-5α-lanosta-8,25-dien-21-oic acid (chagabusone A)		in different doses on various cell lines: A549, H1264, H1299, and Calu-6		21-a, rametenolic acid and chagabusone A showed potent anticancer activity against lung cancer cell lines.	
Inonotus obliquus	Six compounds, 1-lanosterol, 2-3β-hydroxy-8,24-dien-21-al, 3-ergosterol, 4-inotodiol, 5-ergosterol peroxide, 6-trametenolic acid	Breast cancer	PC3 and MDA-MB-231	Variable doses IC_{50} against PC3 as follows: 9.82 ± 0.98 μM for ergosterol, 38.19 ± 1.67 μM for ergosterol peroxide, 63.71 ± 3.31 μM for trametenolic acid, and 73.46 ± 0.64 μM for 3b-hydroxy-8,24-dien-21-al. IC_{50} against MDA-MB-231 as follows: 3b-hydroxy-8,24-dien-21-al (36.50 ± 1.13 μM), ergosterol peroxide (30.23 ± 3.24 μM) and trametenolic acid (55.03 ± 5.40 μM)	Two compounds ergosterol peroxide and trametenolic acid displayed cytotoxicity on both PC3 and MDA-MB-231 cells.	[78]

Lentinula edodes	*Lactripin-1,* apoptosis	Lung cancer	*In vitro*, A549	-	Protein *Lactripin* can induce apoptosis in A549 cells.	[79]
Lentinula edodes	*Lactripin-13 domain,* apoptosis	Lung cancer	*In vitro*, A549	Cell viability and mode of cell death, 20 μg/mL	*Lactripin* 13 domain can induce apoptosis in A549 cells.	[80]
Lentinus squarrosulus Mont.	Partially isolated peptides	Lung cancer	*In vitro*: H460, H292 and H23 cells	-	Both extracts and peptides showed apoptosis due to the reduction of anti-apoptotic Bcl-2 protein and up-regulation of BAX.	[81]
Lentinus squarrosulus	Partially isolated peptides	Lung cancer	*In vitro*: H460, H292 and H23 cells		"Strong suppression on integrin-mediated survival was evidenced with the diminution of integrins and down-stream signals (p-FAK/FAK, p-Src/Src, p-Akt/Akt) consequence with alteration of p53, Bax, Blc-2 and Mcl-1 in cisplatin-treated lung cancer cells".	[82]
Lepista inversa	Clitocine	Tumor	Murine cancer cell lines (3LL) and L1210-tumor-bearing mice.	-	Clitocine showed antitumor activity with a significant increase in lifespan and a decrease in the development of ascites (3 mg/kg).	[83]
Lignosus rhinocerotis (Cooke) Ryvarden (tiger milk mushroom)	Shotgun proteomics and N-terminal protein sequencing	Breast cancer	*In vitro*, MCF7	Fraction 5 (F5) shows two distinct bands with strong cytotoxicity	Phenylmethylsulfonyl fluoride, a specific serine protease inhibitor, inhibited the proteolytic activity. The other	[84]

				against MCF7 (IC_{50} =3.00 ± 1.01 μg/mL)	evidence is the cytotoxicity and cytotoxicity of F5.	
Lignosus rhinocerotis	F5	Breast cancer	MCF7	IC_{50} value of 3μg/mL	Apoptosis induction may involve a cross-talk between the extrinsic and intrinsic apoptotic pathways with upregulation of caspase-8 and -9 activities with a remarkable decrease of Bcl-2.	[85]
Lignosus tigris	Lectins, serine proteases, RNase Gf29 and a 230NA deoxyribonucle-ase	Breast cancer	MCF7-xenograft tumor growth	--	"Selective cytotoxicity via induction of cellular apoptosis by the activation of extrinsic and intrinsic signaling pathways".	[86]
Marasmius oreades	Phenolics (vanillic acid, gallic acid, ferulic acid, and catechin)	Colon and breast cancer	HT-29, MCF-7, and MDA-MB-231	-	Extract has moderate anticancer effect on several cancer cell lines.	[87]
Marasmius oreades	Four chromatogram-phic fractions	Breast cancer	MCF7	-	Affect the process of tumorigenesis through the direct blockage of NF-kappa B activation at the inhibitory protein kappa B level.	[88]
Phellinus linteus	-	Breast cancer	MDA-MB-231 cells	-	Extracts suppressed invasive behavior of breast cancer cells) by inhibiting cell adhesion, migration	[89]

					and invasion (secretion of urokinase-plasminogen activator).	
Phellinus linteus	-	Lung cancer	Mouse and human lung cancer cells	-	Extract regulates cell-cycle arrest and apoptosis (caspase inhibitor Z-VADfmk, DNA fragmentation, caspase activation, and loss of clonogenecity).	[90]
Phellinus linteus	Polysaccharide	Colon cancer	HCT116 and HT29 cells	-	Extracts containing polysaccharide functions in two ways to achieve its lethal synergy with camptothecin 11 in both HCT116 and HT29 cells.	[55]
Phellinus linteus	-	Prostate cancer	PC3 or DU145 cells	Apoptosis in athymic nude mice	*In vivo* study of the extract suggests that it can attenuate tumor growth, as well as cause tumor regression by inducing apoptosis.	[91]
Phellinus linteus	-	Liver cancer	SCID CB-17 mice received a transplant of Hep3B cells	-	Finding suggest that successfully reduction in tumor size, was associated with increase in T cell IL-12, IFN-γ. Other important factors with observed changes includes TNF-α	[92]

					secretion; NK cell activity; and phagocytic ability.	
Phellinus rimosus (Berk) Pilat	-	Breast cancer	Cell line MCF-7, ALB/c mouse model of MCF-7 tumor xenografts	-	The purified protein acts as an immunoregulatory agent to indirectly inhibit malignant proliferation of tumors.	[93]
Pholiota nameko	Water-soluble protein (WSP), MW ~43 kDa	Breast cancer	MCF7	-	WSP inhibited the proliferation by inducing apoptosis. Other observed changes include cell cycle dysruption and the loss of mitochondrial transmembrane potential.	[94]
Pleurotus abalones	Polysaccharide fraction (3.68×10^5 Da)	Breast cancer	MCF-7	MCF-7 (IC_{50} =193 μg/mL).	Treatment causes apoptosis via a mitochondria-mediated pathway. In addition, cell cycle arrest was observed at the S phase and PAP-3 induced up-regulation of p53..	[95]
Pleurotus highking	Purified fraction-III (PEF-III)	Breast cancer	MDA-MB-231 and HCC-1937	Antiproliferative and antimetastatic effects	The antiproliferative and antimigratory effects of PEF-III occur by suppressing Akt signaling. Further, PEF-III significantly decreased the mRNA expression of Ki-67 and MMP-9.	[96]
Pleurotus highking	PEF-III	Breast cancer	MCF-7	Apoptosis	PEF-III has promising anticancer activity, because	[97]

					of induction of apoptosis by a shift in the balance of proapoptotic and antiapoptotic genes.	
Pleurotus nebrodensis	Purified polysaccharides	Several cancer cells	Sarcoma 180 and numerous cell lines (HT-29, NIH3T3, and RAW 264.7)	Mouse sarcoma 180, immunopotentiating properties	Finding suggest that polysaccharides from this mushroom have a potent anticancer effect.	[98]
Pleurotus nebrodensis	Purified polysaccharides	Lung cancer	MRC-5 and A549	A549, apoptosis	Purified polysaccharide can inhibit A549 cell proliferation and induce apoptosis primarily via activating the intrinsic mitochondrial pathway.	[99]
Pleurotus ostreatus	-	Breast and colon cancer	MCF-7 and HT-29 cells	Tumour	Antitumor effect by inducing the expression of the tumor suppressor p53 and the cyclin-dependent kinase inhibitor p21 (CIP1/WAF1), but at the same time inhibiting the phosphorylation of retinoblastoma protein (Rb) in breast and colon cells, respectively.	[100]
Pleurotus ostreatus	-	Blood cancer	KG-1	Hyperthermia	Extract induces apoptosis in KG-1 cells. This comparative study also observed anticancer effects are significantly increased	[101]

					when combined with thermotherapy.	
Poria cocos F.A.Wolf, Synonym-: *Wolfiporia extensa* (Peck) Ginns (1984)	β-glucan PCM3-II	Breast cancer	MCF-7	Immune-enhancing and anti-tumor activities	PCM3-II induced cell-cycle arrest time-dependently at G1 phase, which was associated with downregulation of the unscheduled cyclin D1 and cyclin E expression in MCF-7 cells.	[102]
Cerrena unicolor (CU) and *Pycnoporus sanguineus* (PS)	-	Colon cancer	*In vitro* (HT-29, LS 180, and SW948).	Chemo-preventive activity of bioactive fungal fractions	PS4-II exerted a milder effect on normal CCD 841 CoTr cells than CU1-II	[103]
Ramaria flava	-	Tumor	*In vitro*: several cell lines including MDA-MB-231	MTT assay	Strong antitumor activity against MDA-MB-231 (IC_{50} = 66.54 μg/mL.	[104]
Russula alatoreticula	Phenolic compounds (pyrogallol > cinnamic acid > p-coumaric acid)	Hepatocellular carcinoma	Anticancer property, apoptosis	Hep3B cell (IC_{50} 358.57 μg/mL)	Showed morphological changes, cell cycle arrest, depleting MMP and alleviating ROS through Bax, Bcl2, caspases 3 and 9 intrinsic mitochondrial pathway.	[105]
Schizophyllum commune	Schizophyllan, a β-D-glucan	Breast and liver cancer	Mouse survival, tumor incidence, histopathology, estrogen receptor (ER)	Schizophyllan and tamoxifen reduced the incidence of mammary tumors by 85 and	In both mammary and hepatic carcinomas, tamoxifen was more effective than schizophyllan by the induction of apoptosis.	[106]

			expression, cell proliferation, apoptosis and caspase-3 expression.	75 %, respectively.		
Thelephora aurantiotincta	p-terphenyl derivative, thelephantin O, and vialinin A	Hepatocellular carcinoma	HepG2 and Caco2	-	Ethanol extract has selective cytotoxic agents against cancer cells.	[107]
Taiwanofungus salmoneus	Total phenols, flavonoids, and ergothioneine	Colorectal cancer	Hep-1 and Caco-2		Extracts exhibited better apoptotic effects on Sk-Hep-1 and Caco-2 cells than sorafenib and celecoxib.	[108]
Tricholoma mongolicum	A 66-kDa laccase	Hepatocellular and breast cancer	HepG2 and MCF7	Antiproliferative	Potent antipathogenic protein effective against MCF7 (IC_{50} = 4.2 μM).	[109]
Xylaria schweinitzii	Schweinitzins A and (S)-torosachrysone-8-O-methyl ether	Several type of cancer cells	KB, MCF7, SK-LU-I and HepG2	-	Methanolic extract of *X. schweinitzii* fruiting bodies and its two major constituents Schweinitzins A and (S)-torosachrysone-8-O-methyl ether showed potent anticancer activity.	[110]
MycoPhyto® Complex (MC), *Agaricus blazei, Cordyceps sinensis, Coriolus versicolor, Ganoderma lucidum, Grifola frondosa* and *Polyporus umbellatus*, and β-1,3-glucan from *Saccharomyces cerevisiae*.	-	Breast cancer	Antiproliferative		Mixture of mushroom mycelia, has cytostatic effects by the inhibition of cell proliferation as well as arresting cell cycle.	[111]

Table S5: Summary of active constituents of mushroom(s) and their mechanism of action

Active constituents	Name of the mushroom	Type of cancer	Observation	Mechanism of action	Reference
Beta-(1-3)-glucan	*Agaricus bisporus*	Breast cancer and sarcoma 180-bearing mice	Significantly increased the thymus index and stimulated splenocyte proliferation.	Antitumor activity is related with up-regulation of NK cells activity and expression of IFN-Ɣ in spleen.	[44]
Ergosterol	*Agaricus blazei*	Sarcoma 180-bearing mice, female C57BL/6 mice	Solid tumor (angiogenesis induced direct inhibition)	Ergosterol inhibited the Matrigel-induced neovascularization	[112]
Fraction containing (1-->4)-alpha-D-glucan and (1-->6)-beta-D-glucan branching, compound is HM3-G (molecular mass 380 kDa)	*Agaricus blazei*	Double-grafted tumor system in Balb/c mice	Inhibits tumor cell growth *in vitro* with selective cytotoxicity.	Increasing expression of the Apo2.7 antigen observed on the mitochondrial membranes, so as to induce apoptotic processing	[113]
Fraction-3 (LM-3), containing alpha-1,4-glucan-beta-1,6-glucan complex with an average molecular weight of 20 kDa	*Agaricus blazei*	MethA tumor cell growth	*In vitro*, selective cytotoxicity	Authors predict that the inhibition of the distant tumor may be due to the migration of granulocytes, at the primary tumor site.	[114]
(1,6)-β - D-polyglucose, with an average molecular weight of 10 kDa	*Agaricus blazei*	Mouse tumor	Remarkable tumor regression observed.	-	[115]

Antrocin	*Antrodia camphorata*	Breast cancer	Apoptosis observed with antrocin treatment in MDA-MB-231 cells, (IC_{50} =0.6 μM)	Cleavage of caspase-3 and poly (ADP-ribose) polymerase.	[116]
Antrocin	*Antrodia camphorata*	Breast cancer	MCF-7 and MDA-MB-231, immunomodulatory effects	Transcriptional activation of p53, as established by the fact that ATA failed to induce miR-200c or suppress ZEB1 activity in p53-inhibited cells.	[117]
Polysaccharide peptides (PSP)	*Coriolus versicolor*	Breast cancer	MCF-7, immunomodulatory effects	Polysaccharide peptides exert significant proliferative response of blood lymphocytes, related with IL-6 and IL-1β mRNA upregulation.	[118]
Cordycepin (3'-deoxyadenosine)	*Cordyceps sinensis*	Lung cancer	B16-F1 mouse melanoma cells, hematogenic metastasis	Via blocking of ADP-induced platelet aggregation in mouse model.	[119]
Cordycepin	*Cordyceps sinensis*	Lung cancer	Cell line H1975, apoptosis	Cordycepin inhibits cell proliferation and induces apoptosis via the EGFR signalling pathway.	[120]
Cordycepin	*Cordyceps sinensis*	Lung cancer	NSCLC, apoptosis	Cordycepin is capable of inhibiting NSCLC cell cycle progression as well as inducing apoptosis by interacting with AMP-activated protein kinase (AMPK).	[121]
Cordycepin	*Cordyceps sinensis*	Testicular cancer	MA-10 mouse Leydig tumor cells, cell cycle inhibition	Cordycepin inhibited FGF9-induced testicular tumor growth by suppressing the ERK1/2, Rb/E2F1, expressions of FGFR1-4 proteins and interfering with cell cycle pathways.	[122]
Ergosterol, 5,6-dehydroergosterol and ergosterol peroxide (EP)	*Ganoderma lucidum*	Inflammatory breast cancers (IBC) and other human cancers	*In vitro*, apoptosis	EP shows anti-proliferative effects via arresting G1 phase cell cycle, induced apoptosis via caspase 3/7	[123]

		cell types (solid and blood malignancies)		activation, and PARP cleavage. In addition, EP has invasive effects by inhibiting the expression of total AKT1, AKT2, BCL-XL, Cyclin D1 and c-Myc in IBC cells.	
Ganoderic acid DM	*Ganoderma lucidum*	Breast cancer	*In vitro*, MCF-7 cell proliferation and colony formation	Ganoderic acid induces apoptosis (as observed by DNA fragmentation and cleavage of PARP), as well as decreases the mitochondrial membrane potential.	[124]

Ganoderic acid	*Ganoderma lucidum*	Breast cancer	*In vitro* and *in vivo*, MDA-MB-231, angiogenesis, invasion and apoptosis	Extract induced apoptosis (via suppressing NF-κB activity and the expression profile of its downstream genes), and inhibited proliferation, angiogenesis, invasion .	[125]
Ganoderic acid A	*Ganoderma lucidum*	Breast cancer	*In vitro*, MDA-MB-231, pro-apoptotic function, antineoplastic effect	Extract has effects inhibiting cell viability via JAK2/STAT3 downregulation.	[126]
D-Fraction	*Grifola frondosa*	Tumor-bearing mice	*In vivo*, enhancement of several immune responses	Suppresses progress of tumor, as well as delays the metastatic progress and enhances several immune responses via activation of macrophage, NK and T cells.	[127]
β-glucan	*Grifola frondosa*	Tumors in a murine model	Tumor regression	Soluble β-glucan maitake D-fraction, and cytosine-phosphate-guanine oligodeoxynucleotide synergistically activated dendritic cells (increased the expression of dendritic cell maturation markers and interleukin-12 production).	[128]
D-Fraction, a polysaccharide	*Grifola frondosa*	MM-46 mammary tumor-bearing C3H/HeJ mice	Cytotoxicity against NK-sensitive YAC-1 cells and the expression of CD223	D-Fraction improves toxicity, with production of IL-12 by macrophages.	[129]
α-glucan	*Grifola frondosa*	CT-26 tumor-bearing BALB/c mice and B16 melanoma-bearing C57BL/6 mice	Improvements of immunological parameters	Increased cell proliferation along with increased IFN-γ production by allogeneic $CD4^+$ and $CD8^+$ T cells	[130]

α-glucan	*Grifola frondosa*	Colon-26 carcinoma and B16 melanoma	Improvements of immunological parameters	In the spleen, enhanced immune response by increasing INF-γ-expressing CD4+ and CD8+ cells while INF-γ-expressing CD8+ cells in the tumor lymph nodes. Might be a candidate for immunotherapy against cancer.	[131]
β-glucan	*Grifola frondosa*	Tumors in a murine model	Improvements of immunological parameters	Decrease in the number of immunosuppressive cells (responsible for tumor), increase in the number of infiltrations activated T cells (responsible for tumor), and induction of specific T-cell response (antigen-specific tumor)	[132]
β-(1,3) (1,6)-glucan	*Grifola frondosa*	Murine resident macrophages	Improvements of immunological parameters	Soluble β-glucans induce cell proliferation and cytokine production (through Dectin-1-independent ERK and p38 MAPK activation).	[133]
Heteropolysaccharide	*Grifola frondosa*	BALB/c mice	Induced both therapeutic and preventive effects on colon-26 tumor development.	Could be a potential effective adjuvant to enhance immunotherapy using DC-based vaccination.	[134]
D-Fraction	*Grifola frondosa*	MM46-bearing C3H/HeN mice	Suppression of tumor growth.	D-fraction treatment increased TNF-α and IFN-ϒ (spleen cells) as well as TNF-α expression in NK cells.	[135]
Ergosterol peroxide	*Inonotus obliquus*	Human colorectal cancer	CRC cell lines, anti-proliferative and pro-apoptotic activities	Ergosterol peroxide effectively inhibits colitis-associated colon cancer in AOM/DSS-treated mice. In CRC cells, it down-regulated β-catenin signalling.	[136]

Eburicoic acid	*Laetiporus sulphureus*	Human cancer cell lines (HL-60, SMMC-721, A-549, MCF-7, SW-480)	Exhibited moderate cytotoxicity, IC_{50}s, HL-60=37.5 μM, SMMC-721 = 14.8 μM, A-549 = 15.6 μM, and SW-480 =36.1 μM.	-	[137]
Two mannogalactoglucan-type polysaccharides (WPLE-N-2 and WPLE-A0.5-2)	*Lentinus edodes*	Sarcoma 180 (S-180)-bearing mice	*In vivo*, immunomodulating effects.	β-linkages plays a key role for the antitumor and immunomodulating effects.	[138]
(1→6)-, (1→4)- and (1→3)-linked β-D-glucopyranosyl residues, (1→6)-linked α-D-galactopyranosyl residues, (1→3,6)- and (1→2,4)-linked α-D-mannopyranosyl residues and terminal residues of β-D-glucopyranosyl	*Lentinus edodes*	Sarcoma 180 (S-180)-bearing mice	*In vivo* and *in vitro* (carcinoma HCT-116 and HT-29 cells)	Antitumor bioactivities implanted in Kunming mice.	[139]
Panepoxydone [(1S,5R,6S)-5-hydroxy-3-[(1S)-1-hydroxy-3- methylbut-2-enyl]-7-oxabicyclo [4.1.0] hept-3-en-2-one]	*Lentinus crinitus*	Breast and several other cancer, MCF-7 and TNBC cell lines MDA-MB-231, MDA-MB-468 and MDA-MB-453	Apoptosis and EMT-related proteins expression	Panepoxydone inhibits the NF-kappaB- activated expression of the SEAP	[140,141]
6-Methylheptane-1,2,3,4,5-pentaol, and five ergostanoids	*Lentinus polychrous*	Breast cancer	T47D cells, cell proliferation	Compound exhibited oppressive effect on oestradiol-enhanced cell proliferation while no oestrogenic activity is observed.	[142]
Polysaccharides	*Phellinus linteus*	Colon cancer	Athymic nude mouse SW480 tumor engraft model, cell proliferation	Extract (125-1000 μg/mL) inhibited cell proliferation as well as β-catenin expression.	[143]

Polysaccharides (backbone is α-D-Glc(1→4)-α-D-Glc(1→6) units)	*Phellinus linteus*	Tumor	S-180 sarcoma cells, anti-proliferative effect	Polysaccharide has strong anti-proliferative effect via apoptosis.	[144]
Hispolon	*Phellinus linteus*	Breast and bladder cancer	MCF7, T24 and J8	Hispolon is effective in breast and bladder cancers and downregulates MDM2 via MDM2-recruited activated ERK1/2. Also, hispolon-induced caspase-7 cleavage was inhibited by the ERK1/2 inhibitor, U0126.	[145]
Lectin	*Pholiota adiposa*	Breast cancer	MCF7 and Hep G2 cells, antiproliferative	Antiproliferative activity against Hep G2 cells (2.1μM) and MCF7 cells (3.2μM)	[146]
1- 4 [D-mannitol (C1), ergosta-5,7,22-trien-3β-ol (C2), 5,8- epidioxy-ergosta-6-22-dien-3β-ol (C3), and palmitic acid (C4)	*Pleurotus djamor*	Breast cancer	MDA-MD-231, cytotoxicity	The compound 5,8- epidioxy-ergosta-6-22-dien-3β-ol exhibited better cytotoxic activity than ergosta-5,7,22-trien-3β-ol .	[147]
ergosta-5,7,22-trien-3β-ol (major constituent)	*Pleurotus sajor-caju*	Colorectal cancer, $HTC116^{-p53}$ and $HTC116^{-Bax}$ cells	*In vitro* ($HTC116^{-p53}$ and $HTC116^{-Bax}$ cells), apoptosis-promoting and cell cycle-arrest pathways	p21/p53 cell cycle regulation pathway is probably disrupted by compounds present in hexane extract.	[148]
5alpha,8alpha-Epidioxy-22E-ergosta-6, 22-dien-3beta-ol (ergosterol peroxide)	*Sarcodon aspratus*	Human colorectal cancer, HT29	Suppression of LPS-induced DNA binding activity and phosphorylation inhibition of ERK MAPKs	Ergosterol peroxide suppresses LPS-induced inflammatory responses by inhibiting NF-kappaB and C/EBPbeta transcriptional activity.	[149]
2',3'-dihydroxy-p-terphenyl derivative, thelephantin O	*Thelephora aurantiotincta*	Hepatocellular carcinoma cells	Proliferation suppression via iron chelation	Fe^{++}chelation occurs upstream in the pivotal pathway of 2',3'-dihydroxy-p-terphenyl-induced inhibition of hepatocellular carcinoma cell proliferation.	[150]

Lectin	*Tricholoma mongolicum*	Sarcoma cancer tumour	TML-1 and TML-2 inhibit the growth of implanted sarcoma 180 cells.	In a mouse model both lectins stimulated the production of nitrite ions and activated macrophages.	[151]
Pachymic acid (PA)	*Poria cocos*	Bladder cancer	Apoptopsis, chromatin condensation and DNA fragmentation	A lanostane-type triterpenoid, PA, activates Bid and induces the loss of mitochondrial membrane potential (ΔΨm) with up-regulated pro-apoptotic proteins (Bax and Bad), down-regulated anti-apoptotic proteins (Bcl-2 and Bcl-xL) and cytochrome c release.	[152]
Pachymic acid	*Poria cocos*	Lung cancer	PA induces apoptosis (NCI-H23 and NCI-H460)	PA revealed anti-tumor effects *in vitro* accompanied by induction of G2/M phase arrest and apoptosis through activation of the JNK and ER stress pathways in human lung cancer cells.	[153]
Pachymic acid	*Poria cocos*	Breast cancer	*In vitro* and *in vivo* anticancer activity, MDA-MB-231 (IC_{50} value, 2.13 ± 0.24 μg/mL).	PA has anticancer activity, that could inhibit tumour development via induction of cell apoptosis and G_0/G_1 cell cycle arrest.	[154].

(-) Data not available

Abbreviations

AE- Adverse event
AHCC®-Active hexose correlated compound
FFLZ-Fucose-containing fraction of *G. lucidum*
HCC-Hepatocellular carcinoma
HP- Hematologic parameters
IC50- Half-maximal inhibitory concentration

LOA-Loss of appetite
MDS-Myelodys plastic syndromes
MM- Medicinal mushrooms
OS- Overall survival
QOL-Quality of life
RCT- Randomized, placebo-controlled, double-blind clinical trial
ROS-Reactive oxygen species
SDL-Superfine dispersed lentinan
SPG-Polysaccharide Schizophyllan

References

1. Twardowski, P.; Kanaya, N.; Frankel, P.; Synold, T.; Ruel, C.; Pal, S.K.; Junqueira, M.; Prajapati, M.; Moore, T.; Tryon, P.; Chen, S. A phase I trial of mushroom powder in patients with biochemically recurrent prostate cancer: Roles of cytokines and myeloid-derived suppressor cells for *Agaricus bisporus*-induced prostate-specific antigen responses. *Cancer* **2015**, *121*, 2942–2950, doi:10.1002/cncr.29421.
2. Ahn, W.S.; Kim, D.J.; Chae, G.T.; Lee, J.M.; Bae, S.M.; Sin, J.I.; Kim, Y.W.; Namkoong, S.E.; Lee, I.P. Natural killer cell activity and quality of life were improved by consumption of a mushroom extract, *Agaricus blazei* Murill Kyowa, in gynecological cancer patients undergoing chemotherapy. *Int. J. Gynecol. Cancer* **2004**, *14*, 589–594, doi:10.1111/j.1048-891X.2004.14403.x.
3. Grinde, B.; Hetland, G.; Johnson, E. Effects on gene expression and viral load of a medicinal extract from *Agaricus blazei* in patients with chronic hepatitis C infection. *Int. Immunopharmacol.* **2006**, *6*, 1311–1314, doi:10.1016/j.intimp.2006.04.005.
4. Tangen, J.-M.; Tierens, A.; Caers, J.; Binsfeld, M.; Olstad, O.K.; Trøseid, A.-M.S.; Wang, J.; Tjønnfjord, G.E.; Hetland, G. Clinical Study Immunomodulatory Effects of the *Agaricus blazei* Murrill-Based Mushroom Extract AndoSan in Patients with Multiple Myeloma Undergoing High Dose Chemotherapy and Autologous Stem Cell Transplantation: A Randomized, Double Blinded Clinical Study. **2015**, doi:10.1155/2015/718539.
5. Fortes, R.C.; Lacorte Recôva, V.; Melo, A.L.; Carvalho, R.; Novaes, G.; De Vida, C. Life quality of postsurgical patients with colorectal cancer after supplemented diet with agaricus sylvaticus fungus. *Nutr Hosp* **2010**, *25*, 586–596, doi:10.3305/nh.2010.25.4.4473.
6. Fortes, R.C.; Novaes, M.R.C.G.; Recôva, V.L.; Melo, A.L. Immunological, hematological, and glycemia effects of dietary supplementation with Agaricus sylvaticus on patients' colorectal cancer. *Exp. Biol. Med.* **2009**, *234*, 53–62, doi:10.3181/0806-RM-193.
7. Fortes, R.C; Novaes, M.R.C.G.. The effects of Agaricus sylvaticus fungi dietary supplementation on the metabolism and blood pressure of patients with colorectal cancer during post surgical phase. *Nutr. Hosp.* **2011**, *26*, 176–86.
8. Fortes, R.C.; Recôva, V.L.; Melo, A.L.; Novaes, M.R.C.G. Effects of dietary supplementation with medicinal fungus in fasting glycemia levels of patients with colorectal cancer: A randomized, double-blind, placebo-controlled clinical study. *Nutr. Hosp.* **2008**, *23*, 591–598, doi:10.3305/nutr.
9. Valadares, F.; Novaes, M.R.C.G.; Cañete, R. Effect of *Agaricus sylvaticus* supplementation on nutritional status and adverse events of chemotherapy of breast cancer: A randomized, placebo-controlled, double-blind clinical trial. *Indian J. Pharmacol.* **2013**, *45*, 217–222, doi:10.4103/0253-7613.111894.
10. Tsai, M.Y.; Hung, Y.C.; Chen, Y.H.; Chen, Y.H.; Huang, Y.C.; Kao, C.W.; Su, Y.L.; Chiu, H.H.E.; Rau, K.M. A preliminary randomised controlled study of short-term *Antrodia cinnamomea* treatment combined with chemotherapy for patients with advanced cancer. *BMC Complement. Altern. Med.* **2016**, *16*, doi:10.1186/s12906-016-1312-9.
11. Chay, W.Y.; Tham, C.K.; Toh, H.C.; Lim, H.Y.; Tan, C.K.; Lim, C.; Wang, W.W.; Choo, S.P. *Coriolus versicolor* (Yunzhi) use as therapy in advanced hepatocellular carcinoma patients with poor liver function or who are unfit for standard therapy. *J. Altern. Complement. Med.* **2017**, *23*, 648–652, doi:10.1089/acm.2016.0136.
12. Torkelson, C.J.; Sweet, E.; Martzen, M.R.; Sasagawa, M.; Wenner, C.A.; Gay, J.; Putiri, A.; Standish, L.J. Phase 1 clinical trial of trametes versicolor in women with breast cancer . *ISRN Oncol.* **2012**, *2012*, 1–7, doi:10.5402/2012/251632.
13. Zhao, H.; Zhang, Q.; Zhao, L.; Huang, X.; Wang, J.; Kang, X. Spore powder of *Ganoderma lucidum* improves cancer-related fatigue in breast cancer patients undergoing endocrine therapy: A pilot clinical trial. *Evidence-based Complement. Altern. Med.* **2012**, *2012*, doi:10.1155/2012/809614.
14. Griessmayr, P.C.; Gauthier, M.; Barber, L.G.; Cotter, S.M. Mushroom-derived Maitake PETfraction as single agent for the treatment of lymphoma in dogs. *J. Vet. Intern. Med.* **2007**, *21*, 1409–1412, doi:10.1892/07-070.1.
15. Deng, G.; Lin, H.; Seidman, A.; Fornier, M.; D'Andrea, G.; Wesa, K.; Yeung, S.; Cunningham-

Rundles, S.; Vickers, A.J.; Cassileth, B. A phase I/II trial of a polysaccharide extract from *Grifola frondosa* (Maitake mushroom) in breast cancer patients: Immunological effects. *J. Cancer Res. Clin. Oncol.* **2009**, *135*, 1215–1221, doi:10.1007/s00432-009-0562-z.

16. Wesa, K.M.; Cunningham-Rundles, S.; Klimek, V.M.; Vertosick, E.; Coleton, M.I.; Yeung, K.S.; Lin, H.; Nimer, S.; Cassileth, B.R. Maitake mushroom extract in myelodysplastic syndromes (MDS): a phase II study. *Cancer Immunol. Immunother.* **2014**, *64*, 237–247, doi:10.1007/s00262-014-1628-6.

17. Sumiyoshi, Y.; Hashine, K.; Kakehi, Y.; Yoshimura, K.; Satou, T.; Kuruma, H.; Namiki, S.; Shinohara, N. Dietary administration of mushroom mycelium extracts in patients with early stage prostate cancers managed expectantly: A phase II study. *Jpn. J. Clin. Oncol.* **2010**, *40*, 967–972, doi:10.1093/jjco/hyq081.

18. Yamaguchi, Y.; Miyahara, E.; Hihara, J. Efficacy and safety of orally administered lentinula edodes mycelia extract for patients undergoing cancer chemotherapy: A pilot study. *Am. J. Chin. Med.* **2011**, *39*, 451–459, doi:10.1142/S0192415X11008956.

19. DeVere White, R.W.; Hackman, R.M.; Soares, S.E.; Beckett, L.A.; Sun, B. Effects of a mushroom mycelium extract on the treatment of prostate cancer. *Urology* **2002**, *60*, 640–644, doi:10.1016/S0090-4295(02)01856-3.

20. Ito, T.; Urushima, H.; Sakaue, M.; Yukawa, S.; Honda, H.; Hirai, K.; Igura, T.; Hayashi, N.; Maeda, K.; Kitagawa, T.; et al. Reduction of adverse effects by a mushroom product, active hexose correlated compound (AHCC) in patients with advanced cancer during chemotherapy-the significance of the levels of HHV-6 DNA in saliva as a surrogate biomarker during chemotherapy. *Nutr. Cancer* **2014**, *66*, 377–382, doi:10.1080/01635581.2014.884232.

21. Zhou, D.; Lin, L. Effect of Jinshuibao capsule on the immunological function of 36 patients with advanced cancer. *Zhongguo Zhong Xi Yi Jie He Za Zhi .* **1995**, *15*, 476–478.

22. Gao, Y.; Tang, W.; Dai, X.; Gao, H.; Chen, G.; Ye, J.; Chan, E.; Koh, H.L.; Li, X.; Zhou, S. Effects of water-soluble *Ganoderma lucidum* polysaccharides on the immune functions of patients with advanced lung cancer. *J. Med. Food* **2005**, *8*, 159–168, doi:10.1089/jmf.2005.8.159.

23. Gao, Y.; Zhou, S.; Jiang, W.; Huang, M.; Dai, X. Effects of Ganopoly® (a *Ganoderma lucidum* polysaccharide extract) on the immune functions in advanced-stage cancer patients. *Immunol. Invest.* **2003**, *32*, 201–215, doi:10.1081/IMM-120022979.

24. Gao, Y.; Dai, X.; Chen, G.; Ye, J.; Zhou, S. A Randomized, placebo-controlled, multicenter study of *Ganoderma lucidum* (W.Curt.:Fr.) Lloyd (Aphyllophoromycetideae) polysaccharides (Ganopoly) in patients with advanced lung cancer. *Int. J. Med. Mushrooms* **2003**, *5*, 14, doi:10.1615/interjmedicmush.v5.i4.40.

25. Mo H; Hong M; Zhang J Effect of Wuse - Lingzhi - Jiaonang on reducing side-effects of radiotherapy and improving immune function in patients with nasopharyngeal cancer — MD Anderson Cancer Center. *Chinese J. Clin. Oncol.* **1999**, *26*, 216–218.

26. Oka S; Tanaka S; Yoshida S; Hiyama T; Ueno Y; Ito M; et al. A water-soluble extract from culture medium of Ganoderma lucidum mycelia suppresses the development of colorectal adenomas *Hiroshima J Med Sci .* **2010**, *59*, 1–6.

27. Nakano, H.; Namatame, K.; Nemoto, H.; Motohashi, H.; Nishiyama, K.; Kumada, K. A multi-institutional prospective study of lentinan in advanced gastric cancer patients with unresectable and recurrent diseases: Effect on prolongation of survival and improvement of quality of life. *Hepatogastroenterology.* **1999**, *46*, 2662–2668.

28. Hazama, S.; Watanabe, S.; Ohashi, M.; Yagi, M.; SuzukI, M.; Matsuda, K.; Yamamoto, T.; Suga, Y.; Suga, T.; Nakazawa, S.; Oka, M. Efficacy of Orally Administered Superfine Dispersed Lentinan (β-1,3-Glucan) for the Treatment of Advanced Colorectal Cancer. *Anticancer Res.* **2009**, *29*.

29. Yang, P.; Liang, M.; Zhang, Y.; Shen, B. Clinical application of a combination therapy of lentinan, multi-electrode RFA and TACE in HCC. *Adv. Ther.* **2008**, *25*, 787–794, doi:10.1007/s12325-008-0079-x.

30. Isoda, N.; Eguchi, Y.; Nukaya, H.; Hosho, K.; Suga, Y.; Sugae, T.; Nakazawa, S.; Sugano, K. Clinical efficacy of superfine dispersed lentinan (β-1,3-glucan) in patients with hepatocellular

carcinoma. *Hepatogastroenterology.* **2009**, *56*, 437–441.

31. Okamura, K.; Suzuki, M.; Yajima, A.; Chihara, T.; Fujiwara, A.; Fukuda, T.; Goto, S.; Ichinohe, K.; Jimi, S.; Kasamatsu, T.; Kawai, N., Clinical evaluation of Schizophyllan combined with irradiation in patients with cervical cancer: A randomized controlled study. *Cancer* **1986**, *58*, 865–872.

32. Zuo, Z.; Zuo, Z.; Liying, Z. Clinical observation on alleviating chemotherapy's side effect of psp in treating gastric careinoma. *LIAONING J. Tradit. Chinese Med.* **2001**, *28*, 668–669.

33. Chen, S.; Oh, S.R.; Phung, S.; Hur, G.; Ye, J.J.; Kwok, S.L.; Shrode, G.E.; Belury, M.; Adams, L.S.; Williams, D. Anti-aromatase activity of phytochemicals in white button mushrooms (A*garicus bisporus*). *Cancer Res.* **2006**, *66*, 12026–12034, doi:10.1158/0008-5472.CAN-06-2206.

34. Yu L; Fernig DG; Smith JA; Milton JD; Rhodes JM Reversible inhibition of proliferation of epithelial cell lines by *Agaricus bisporus* (edible mushroom) lectin. *Cancer Res.* **1993**, *53*, 4627–4632.

35. Rachmawati, H.; Sundari, S.; Nabila, N.; Tandrasasmita, O.M.; Amalia, R.; Siahaan, T.J.; Tjandrawinata, R.R.; Ismaya, W.T. Orf239342 from the mushroom *Agaricus bisporus* is a mannose binding protein. *Biochem. Biophys. Res. Commun.* **2019**, *515*, 99–103, doi:10.1016/j.bbrc.2019.05.107.

36. Cheung, Y.H.; Sheridan, C.M.; Lo, A.C.Y.; Lai, W.W. Lectin from *Agaricus bisporus* inhibited S phase cell population and Akt phosphorylation in human RPE cells. *Investig. Ophthalmol. Vis. Sci.* **2012**, *53*, 7469–7475, doi:10.1167/iovs.12-10589.

37. Sun, Y.; Cheng, M.; Dong, L.; Yang, K.; Ma, Z.; Yu, S.; Yan, P.; Bai, K.; Zhu, X.; Zhang, Q. *Agaricus blazei* extract (FA-2-b-β) induces apoptosis in chronic myeloid leukemia cells. *Oncol. Lett.* **2020**, *20*, doi:10.3892/ol.2020.12133.

38. Shimizu, T.; Kawai, J.; Ouchi, K.; Kikuchi, H.; Osima, Y.; Hidemi, R. Agarol, an ergosterol derivative from Agaricus blazei, induces caspase-independent apoptosis in human cancer cells. *Int. J. Oncol.* **2016**, *48*, 1670–1678, doi:10.3892/ijo.2016.3391.

39. Liu, Y.; Zhang, L.; Zhu, X.; Wang, Y.; Liu, W.; Gong, W. Polysaccharide Agaricus blazei Murill stimulates myeloid derived suppressor cell differentiation from M2 to M1 type, which mediates inhibition of tumour immune-evasion via the Toll-like receptor 2 pathway. *Immunology* **2015**, *146*, 379–391, doi:10.1111/imm.12508.

40. Kobayashi, H.; Yoshida, R.; Kanada, Y.; Fukuda, Y.; Yagyu, T.; Inagaki, K.; Kondo, T.; Kurita, N.; Suzuki, M.; Kanayama, N.; et al. Suppressing effects of daily oral supplementation of beta-glucan extracted from *Agaricus blazei* Murill on spontaneous and peritoneal disseminated metastasis in mouse model. *J. Cancer Res. Clin. Oncol.* **2005**, *131*, 527–538, doi:10.1007/s00432-005-0672-1.

41. Akiyama, H.; Endo, M.; Matsui, T.; Katsuda, I.; Emi, N.; Kawamoto, Y.; Koike, T.; Beppu, H. Agaritine from *Agaricus blazei* Murrill induces apoptosis in the leukemic cell line U937. *Biochim. Biophys. Acta - Gen. Subj.* **2011**, *1810*, 519–525, doi:10.1016/j.bbagen.2011.02.010.

42. Cheng, F.; Yan, X.; Zhang, M.; Chang, M.; Yun, S.; Meng, J.; Liu, J.; Feng, C.P. Regulation of RAW 264.7 cell-mediated immunity by polysaccharides from: *Agaricus blazei* Murill via the MAPK signal transduction pathway. *Food Funct.* **2017**, *8*, 1475–1480, doi:10.1039/c6fo01332e.

43. Ito H, S.K. Antitumor effects of a new polysaccharide-protein complex (ATOM) prepared from *Agaricus blazei* (Iwade strain 101) "Himematsutake" and its mechanisms in tumor-bearing mice - PubMed. *Anticancer Res.* **1997**, *17*, 277–284.

44. Niu, Y.C.; Liu, J.C.; Zhao, X.M.; Cao, J. A low molecular weight polysaccharide isolated from *Agaricus blazei* Murill (LMPAB) exhibits its anti-metastatic effect by down-regulating metalloproteinase-9 and up-regulating Nm23-H1. *Am. J. Chin. Med.* **2009**, *37*, 909–921, doi:10.1142/S0192415X09007351.

45. Gonzaga, M.L.C.; Bezerra, D.P.; Alves, A.P.N.N.; De Alencar, N.M.N.; De Oliveira Mesquita, R.; Lima, M.W.; De Aguiar Soares, S.; Pessoa, C.; De Moraes, M.O.; Costa-Lotufo, L.V. In vivo growth-inhibition of Sarcoma 180 by an α-(1 → 4)-glucan-β-(1 → 6)-glucan-protein complex polysaccharide obtained from *Agaricus blazei* Murill. *J. Nat. Med.* **2009**, *63*, 32–40, doi:10.1007/s11418-008-0286-4.

46. Da Silva, A.F.; Sartori, D.; MacEdo, F.C.; Ribeiro, L.R.; Fungaro, M.H.P.; Mantovani, M.S. Effects

of β-glucan extracted from *Agaricus blazei* on the expression of ERCC5, CASP9, and CYP1A1 genes and metabolic profile in HepG2 cells. *Hum. Exp. Toxicol.* **2013**, *32*, 647–654, doi:10.1177/0960327112468173.

47. Murakawa, K.; Fukunaga, K.; Tanouchi, M.; Hosokawa, M.; Hossain, Z.; Takahashi, K. Therapy of myeloma in vivo using marine phospholipid in combination with *Agaricus blazei* Murill as an immune respond activator. *J. Oleo Sci.* **2007**, *56*, 179–188, doi:10.5650/jos.56.179.

48. Kimura, Y.; Kido, T.; Takaku, T.; Sumiyoshi, M.; Baba, K. Isolation of an anti-angiogenic substance from *Agaricus blazei* Murill: Its antitumor and antimetastatic actions. *Cancer Sci.* **2004**, *95*, 758–764, doi:10.1111/j.1349-7006.2004.tb03258.x.

49. Taveira, V.C.; Novaes, M.R.C.G.; Reis, M.D.A.; Da Silva, M.F. Hematologic and metabolic effects of dietary supplementation with Agaricus sylvaticus fungi on rats bearing solid walker 256 tumor. *Exp. Biol. Med.* **2008**, *233*, 1341–1347, doi:10.3181/0803-RM-107.

50. Jiao, C.; Xie, Y.Z.; Yang, X.; Li, H.; Li, X.M.; Pan, H.H.; Cai, M.H.; Zhong, H.M.; Yang, B.B. Anticancer activity of *Amauroderma rude*. *PLoS One* **2013**, *8*, doi:10.1371/journal.pone.0066504.

51. Yang, H.L.; Kuo, Y.H.; Tsai, C.T.; Huang, Y.T.; Chen, S.C.; Chang, H.W.; Lin, E.; Lin, W.H.; Hseu, Y.C. Anti-metastatic activities of *Antrodia camphorata* against human breast cancer cells mediated through suppression of the MAPK signaling pathway. *Food Chem. Toxicol.* **2011**, *49*, 290–298, doi:10.1016/j.fct.2010.10.031.

52. Lee, C.C.; Yang, H.L.; Way, T. Der; Kumar, K.J.S.; Juan, Y.C.; Cho, H.J.; Lin, K.Y.; Hsu, L.S.; Chen, S.C.; Hseu, Y.C. Inhibition of cell growth and induction of apoptosis by Antrodia camphorata in HER-2/neu-overexpressing breast cancer cells through the induction of ROS, depletion of HER-2/neu, and disruption of the PI3K/Akt signaling pathway. *Evidence-based Complement. Altern. Med.* **2012**, *2012*, doi:10.1155/2012/702857.

53. Chen, Y.Y.; Chou, P.Y.; Chien, Y.C.; Wu, C.H.; Wu, T.S.; Sheu, M.J. Ethanol extracts of fruiting bodies of *Antrodia cinnamomea* exhibit anti-migration action in human adenocarcinoma CL1-0 cells through the MAPK and PI3K/AKT signaling pathways. *Phytomedicine* **2012**, *19*, 768–778, doi:10.1016/j.phymed.2012.02.016.

54. Chen, M.C.; Hsu, W.L.; Chou, T.C. Anti-cachectic effect of *Antrodia cinnamomea* extract in lung tumor-bearing mice under chemotherapy. *Oncotarget* **2018**, *9*, 19584–19596, doi:10.18632/oncotarget.24680.

55. Lin, Y.S.; Lin, Y.Y.; Yang, Y.H.; Lin, C.L.; Kuan, F.C.; Lu, C.N.; Chang, G.H.; Tsai, M.S.; Hsu, C.M.; Yeh, R.A.; et al. *Antrodia cinnamomea* extract inhibits the proliferation of tamoxifen-resistant breast cancer cells through apoptosis and skp2/microRNAs pathway. *BMC Complement. Altern. Med.* **2018**, *18*, doi:10.1186/s12906-018-2204-y.

56. Du, Y.C.; Chang, F.R.; Wu, T.Y.; Hsu, Y.M.; El-Shazly, M.; Chen, C.F.; Sung, P.J.; Lin, Y.Y.; Lin, Y.H.; Wu, Y.C.; et al. Antileukemia component, dehydroeburicoic acid from *Antrodia camphorata* induces DNA damage and apoptosis in vitro and in vivo models. *Phytomedicine* **2012**, *19*, 788–796, doi:10.1016/j.phymed.2012.03.014.

57. Chang, C.T.; Korivi, M.; Huang, H.C.; Thiyagarajan, V.; Lin, K.Y.; Huang, P.J.; Liu, J.Y.; Hseu, Y.C.; Yang, H.L. Inhibition of ROS production, autophagy or apoptosis signaling reversed the anticancer properties of Antrodia salmonea in triple-negative breast cancer (MDA-MB-231) cells. *Food Chem. Toxicol.* **2017**, *103*, 1–17, doi:10.1016/j.fct.2017.02.019.

58. Chang, C.T.; Hseu, Y.C.; Thiyagarajan, V.; Huang, H.C.; Hsu, L.S.; Huang, P.J.; Liu, J.Y.; Liao, J.W.; Yang, H.L. *Antrodia salmonea* induces G2 cell-cycle arrest in human triple-negative breast cancer (MDA-MB-231) cells and suppresses tumor growth in athymic nude mice. *J. Ethnopharmacol.* **2017**, *196*, 9–19, doi:10.1016/j.jep.2016.12.018.

59. Hseu, Y.C.; Lin, Y.C.; Rajendran, P.; Thigarajan, V.; Mathew, D.C.; Lin, K.Y.; Way, T. Der; Liao, J.W.; Yang, H.L. *Antrodia salmonea* suppresses invasion and metastasis in triple-negative breast cancer cells by reversing EMT through the NF-κB and Wnt/β-catenin signaling pathway. *Food Chem. Toxicol.* **2019**, *124*, 219–230, doi:10.1016/j.fct.2018.12.009.

60. Yang, H.L.; Lin, R.W.; Rajendran, P.; Mathew, D.C.; Thigarajan, V.; Lee, C.C.; Hsu, C.J.; Hseu, Y.C. *Antrodia salmonea*-induced oxidative stress abrogates HER-2 signaling cascade and enhanced apoptosis in ovarian carcinoma cells. *J. Cell. Physiol.* **2019**, *234*, 3029–3042,

doi:10.1002/jcp.27123.

61. Yang, H.-L.; Liu, H.-W.; Shrestha, S.; Thiyagarajan, V.; Huang, H.-C.; Hseu, Y.-C. *Antrodia salmonea* induces apoptosis and enhances cytoprotective autophagy in colon cancer cells. *Aging (Albany. NY).* **2021**, *13*, doi:10.18632/aging.203019.

62. Pan, H.; Han, Y.; Huang, J.; Yu, X.; Jiao, C.; Yang, X.; Dhaliwal, P.; Xie, Y.; Yang, B.B. Purification and identification of a polysaccharide from medicinal mushroom Amauroderma rude with immunomodulatory activity and inhibitory effect on tumor growth. *Oncotarget* **2015**, *6*, 17777–17791, doi:10.18632/oncotarget.4397.

63. Li, X.; Wu, Q.; Xie, Y.; Ding, Y.; Du, W.W.; Sdiri, M.; Yang, B.B. Ergosterol purified from medicinal mushroom Amauroderma rude inhibits cancer growth in vitro and in vivo by up-regulating multiple tumor suppressors. *Oncotarget* **2015**, *6*, 17832–17846, doi:10.18632/oncotarget.4026.

64. Ping, Z.; Xu, H.; Liu, T.; Huang, J.; Meng, Y.; Xu, X.; Li, W.; Zhang, L. Anti-hepatoma activity of the stiff branched β-d-glucan and effects of molecular weight. *J. Mater. Chem. B* **2016**, *4*, 4565–4573, doi:10.1039/c6tb01299j.

65. Matuszewska, A.; Stefaniuk, D.; Jaszek, M.; Pięt, M.; Zając, A.; Matuszewski, Ł.; Cios, I.; Grąz, M.; Paduch, R.; Bancerz, R. Antitumor potential of new low molecular weight antioxidative preparations from the white rot fungus Cerrena unicolor against human colon cancer cells. *Sci. Rep.* **2019**, *9*, doi:10.1038/s41598-018-37947-z.

66. Wu, J.Y.; Zhang, Q.X.; Leung, P.H. Inhibitory effects of ethyl acetate extract of Cordyceps sinensis mycelium on various cancer cells in culture and B16 melanoma in C57BL/6 mice. *Phytomedicine* **2007**, *14*, 43–49, doi:10.1016/j.phymed.2005.11.005.

67. Ho, C.Y.; Kim, C.F.; Leung, K.N.; Fung, K.P.; Tse, T.F.; Chan, H.; Lau, C.B.S. Differential anti-tumor activity of Coriolus versicolor (Yunzhi) extract through p53- and/or Bcl-2-dependent apoptotic pathway in human breast cancer cells. *Cancer Biol. Ther.* **2005**, *4*, 638–644, doi:10.4161/cbt.4.6.1721.

68. Luo, K.W.; Yue, G.G.L.; Ko, C.H.; Lee, J.K.M.; Gao, S.; Li, L.F.; Li, G.; Fung, K.P.; Leung, P.C.; Lau, C.B.S. In vivo and in vitro anti-tumor and anti-metastasis effects of *Coriolus versicolor* aqueous extract on mouse mammary 4T1 carcinoma. *Phytomedicine* **2014**, *21*, 1078–1087, doi:10.1016/j.phymed.2014.04.020.

69. Torres, S.; Cajas, D.; Palfner, G.; Astuya, A.; Aballay, A.; Pérez, C.; Hernández, V.; Becerra, J. Steroidal composition and cytotoxic activity from fruiting body of *Cortinarius xiphidipus*. *Nat. Prod. Res.* **2017**, *31*, 473–476, doi:10.1080/14786419.2016.1185717.

70. Maruyama, H.; Ikekawa, T. Immunomodulation and antitumor activity of a mushroom product, proflamin, isolated from *Flammulina velutipes* (W. Curt.: Fr.) singer (agaricomycetideae). *Int. J. Med. Mushrooms* **2007**, *9*, 109–122, doi:10.1615/IntJMedMushr.v9.i2.20.

71. YC, C.; YM, H.; MF, W.; CC, O.; YW, L.; KH, L.; JL, K. Interruption of lung cancer cell migration and proliferation by fungal immunomodulatory protein FIP-fve from *Flammulina velutipes*. *J. Agric. Food Chem.* **2013**, *61*, 12044–12052, doi:10.1021/JF4030272.

72. Deveci, E.; Tel-Çayan, G.; Duru, M.E.; Öztürk, M. Isolation, characterization, and bioactivities of compounds from *Fuscoporia torulosa* mushroom. *J. Food Biochem.* **2019**, *43*, doi:10.1111/jfbc.13074.

73. Wang, C.; Shi, S.; Chen, Q.; Lin, S.; Wang, R.; Wang, S.; Chen, C. Antitumor and Immunomodulatory Activities of *Ganoderma lucidum* polysaccharides in Glioma-Bearing Rats. *Integr. Cancer Ther.* **2018**, *17*, 674–683, doi:10.1177/1534735418762537.

74. Qu, L.; Li, S.; Zhuo, Y.; Chen, J.; Qin, X.; Guo, G. Anticancer effect of triterpenes from *Ganoderma lucidum* in human prostate cancer cells. *Oncol. Lett.* **2017**, *14*, 7467–7472, doi:10.3892/ol.2017.7153.

75. Zhao, R. lin; He, Y. min Network pharmacology analysis of the anti-cancer pharmacological mechanisms of Ganoderma lucidum extract with experimental support using Hepa1-6-bearing C57 BL/6 mice. *J. Ethnopharmacol.* **2018**, *210*, 287–295, doi:10.1016/j.jep.2017.08.041.

76. Soares, R.; Meireles, M.; Rocha, A.; Pirraco, A.; Obiol, D.; Alonso, E.; Joos, G.; Balogh, G. Maitake (D fraction) mushroom extract induces apoptosis in breast cancer cells by BAK-1 gene activation. *J. Med. Food* **2011**, *14*, 563–572, doi:10.1089/JMF.2010.0095.

77. Baek, J.; Roh, H.S.; Baek, K.H.; Lee, S.; Lee, S.; Song, S.S.; Kim, K.H. Bioactivity-based analysis and chemical characterization of cytotoxic constituents from Chaga mushroom (Inonotus obliquus) that induce apoptosis in human lung adenocarcinoma cells. *J. Ethnopharmacol.* **2018**, *224*, 63–75, doi:10.1016/j.jep.2018.05.025.

78. Ma, L.; Chen, H.; Dong, P.; Lu, X. Anti-inflammatory and anticancer activities of extracts and compounds from the mushroom *Inonotus obliquus*. *Food Chem.* **2013**, *139*, 503–508, doi:10.1016/j.foodchem.2013.01.030.

79. Liu, B.; Zhong, M.; Lun, Y.; Wang, X.; Sun, W.; Li, X.; Ning, A.; Cao, J.; Zhang, W.; Liu, L.; Huang, M. A novel apoptosis correlated molecule: expression and characterization of protein Latcripin-1 from *Lentinula edodes* C(91-3). *Int. J. Mol. Sci.* **2012**, *13*, 6246–6265, doi:10.3390/IJMS13056246.

80. Wang, J.; Zhong, M.; Liu, B.; Sha, L.; Lun, Y.; Zhang, W.; Li, X.; Wang, X.; Cao, J.; Ning, A.; Huang, M.J. Expression and functional analysis of novel molecule - Latcripin-13 domain from *Lentinula edodes* C91-3 produced in prokaryotic expression system. *Gene* **2015**, *555*, 469–475, doi:10.1016/J.GENE.2014.11.014.

81. Prateep, A.; Sumkhemthong, S.; Suksomtip, M.; Chanvorachote, P.; Chaotham, C. Peptides extracted from edible mushroom: *Lentinus squarrosulus* induces apoptosis in human lung cancer cells. *Pharm. Biol.* **2017**, *55*, 1792–1799, doi:10.1080/13880209.2017.1325913.

82. Khine, H.E.E.; Ecoy, G.A.U.; Roytrakul, S.; Phaonakrop, N.; Pornputtapong, N.; Prompetchara, E.; Chanvorachote, P.; Chaotham, C. Chemosensitizing activity of peptide from *Lentinus squarrosulus* (Mont.) on cisplatin-induced apoptosis in human lung cancer cells. *Sci. Rep.* **2021**, *11*, doi:10.1038/s41598-021-83606-1.

83. Fortin, H.; Tomasi, S.; Delcros, J.G.; Bansard, J.Y.; Boustie, J. In vivo antitumor activity of clitocine, an exocyclic amino nucleoside isolated from *Lepista inversa*. *ChemMedChem* **2006**, *1*, 189–196, doi:10.1002/CMDC.200500029.

84. Yap, H.Y.Y.; Fung, S.Y.; Ng, S.T.; Tan, C.S.; Tan, N.H. Shotgun proteomic analysis of tiger milk mushroom (L*ignosus rhinocerotis*) and the isolation of a cytotoxic fungal serine protease from its sclerotium. *J. Ethnopharmacol.* **2015**, *174*, 437–451, doi:10.1016/j.jep.2015.08.042.

85. Yap, H.Y.Y.; Tan, N.H.; Ng, S.T.; Tan, C.S.; Fung, S.Y. Molecular attributes and apoptosisinducing activities of a putative serine protease isolated from Tiger Milk mushroom (*Lignosus rhinocerus*) sclerotium against breast cancer cells in vitro. *PeerJ* **2018**, *2018*, doi:10.7717/peerj.4940.

86. Kong, B.H.; Teoh, K.H.; Tan, N.H.; Tan, C.S.; Ng, S.T.; Fung, S.Y. Proteins from *Lignosus tigris* with selective apoptotic cytotoxicity towards MCF7 cell line and suppresses MCF7- xenograft tumor growth. *PeerJ* **2020**, *8*, doi:10.7717/peerj.9650.

87. Shomali, N.; Onar, O.; Karaca, B.; Demirtas, N.; Cihan, A.C.; Akata, I.; Yildirim, O. Antioxidant, anticancer, antimicrobial, and antibiofilm properties of the culinary-medicinal fairy ring mushroom, *Marasmius oreades* (Agaricomycetes). *Int. J. Med. Mushrooms* **2019**, *21*, 571–582, doi:10.1615/INTJMEDMUSHROOMS.2019030874.

88. Petrova, R.D.; Mahajna, J.; Wasser, S.P.; Ruimi, N.; Denchev, C.M.; Sussan, S.; Nevo, E.; Reznick A.Z. Marasmius oreades substances block NF-kappaB activity through interference with IKK activation pathway. *Mol. Biol. Rep.* **2009**, *36*, 737–744, doi:10.1007/S11033-008-9237-0.

89. Sliva, D.; Jedinak, A.; Kawasaki, J.; Harvey, K.; Slivova, V. *Phellinus linteus* suppresses growth, angiogenesis and invasive behaviour of breast cancer cells through the inhibition of AKT signalling. *Br. J. Cancer* **2008**, *98*, 1348–1356, doi:10.1038/SJ.BJC.6604319.

90. Guo, J.; Zhu, T.; Collins, L.; Xiao, Z.X.; Kim, S.H.; Chen, C.Y. Modulation of lung cancer growth arrest and apoptosis by *Phellinus linteus*. *Mol. Carcinog.* **2007**, *46*, 144–154, doi:10.1002/MC.20275.

91. Tsuji, T.; Du, W.; Nishioka, T.; Chen, L.; Yamamoto, D.; Chen, C.Y. *Phellinus linteus* extract sensitizes advanced prostate cancer cells to apoptosis in athymic nude mice. *PLoS One* **2010**, *5*, doi:10.1371/JOURNAL.PONE.0009885.

92. Huang, H.Y.; Chieh, S.Y.; Tso, T.K.; Chien, T.Y.; Lin, H.T.; Tsai, Y.C. Orally administered mycelial culture of *Phellinus linteus* exhibits antitumor effects in hepatoma cell-bearing mice. *J. Ethnopharmacol.* **2011**, *133*, 460–466, doi:10.1016/J.JEP.2010.10.015.

93. Zhang, Y.; Zhang, Y.; Gao, W.; Zhou, R.; Liu, F.; Ng, T.B. A novel antitumor protein from the

mushroom *Pholiota nameko* induces apoptosis of human breast adenocarcinoma MCF-7 cells in vivo and modulates cytokine secretion in mice bearing MCF-7 xenografts. *Int. J. Biol. Macromol.* **2020**, *164*, 3171–3178, doi:10.1016/J.IJBIOMAC.2020.08.187.

94. Qian, L.; Zhang, Y.; Liu, F. Purification and characterization of a ~43 kDa antioxidant protein with antitumor activity from *Pholiota nameko*. *J. Sci. Food Agric.* **2016**, *96*, 1044–1052, doi:10.1002/JSFA.7194.

95. Shi, X.; Zhao, Y.; Jiao, Y.; Shi, T.; Yang, X. ROS-dependent mitochondria molecular mechanisms underlying antitumor activity of *Pleurotus abalonus* acidic polysaccharides in human breast cancer MCF-7 cells. *PLoS One* **2013**, *8*, doi:10.1371/JOURNAL.PONE.0064266.

96. Haque, M.A.; Reza, A.S.M.A.; Nasrin, M.S.; Rahman, M.A. *Pleurotus highking* mushrooms potentiate antiproliferative and antimigratory activity against triple-negative breast cancer cells by suppressing Akt signaling. *Integr. Cancer Ther.* **2020**, *19*, doi:10.1177/1534735420969809.

97. Ma, H.; Mau, I.. *Pleurotus highking* mushroom induces apoptosis by altering the balance of proapoptotic and antiapoptotic genes in breast cancer cells and inhibits tumor sphere formation. *Medicina (Kaunas).* **2019**, *55*, doi:10.3390/MEDICINA55110716.

98. Cha, Y.J.; Alam, N.; Lee, J.S.; Lee, K.R.; Shim, M.J.; Lee, M.W.; Kim, H.Y.; Shin, P.G.; Cheong, J.C.; Yoo, Y.B.; Lee, T.S. Anticancer and immunopotentiating activities of crude polysaccharides from *Pleurotus nebrodensis* on mouse sarcoma 180. *Mycobiology* **2012**, *40*, 236–243, doi:10.5941/MYCO.2012.40.4.236.

99. Cui, H.; Wang, C.; Wang, Y.; Li, Z.; Zhang, Y.; Chen, M.; Li, F. *Pleurotus nebrodensis* polysaccharide induces apoptosis in human non-small cell lung cancer A549 cells. *Carbohydr. Polym.* **2014**, *104*, 246–252, doi:10.1016/J.CARBPOL.2014.01.001.

100. Andrej, J.; Silva, D. *Pleurotus ostreatus* inhibits proliferation of human breast and colon cancer cells through p53-dependent as well as p53-independent pathway. *Int J Oncol.* **2008**, *33*, 1307–1313.

101. Ebrahimi, A.; Atashi, A.; Soleimani, M.; Mashhadikhan, M.; Barahimi, A.; Kaviani, S. Comparison of anticancer effect of *Pleurotus ostreatus* extract with doxorubicin hydrochloride alone and plus thermotherapy on erythroleukemia cell line. *J. Complement. Integr. Med.* **2017**, *15*, doi:10.1515/JCIM-2016-0136.

102. Zhang, M.; Chiu, L.; Cheung, P.; Ooi, V. Growth-inhibitory effects of a beta-glucan from the mycelium of Poria cocos on human breast carcinoma MCF-7 cells: cell-cycle arrest and apoptosis induction - PubMed. *Oncol Rep .* **2006**, *15*, 637–643.

103. Pięt, M.; Zając, A.; Paduch, R.; Jaszek, M.; Frant, M.; Stefaniuk, D.; Matuszewska, A.; Grzywnowicz, K. Chemopreventive activity of bioactive fungal fractions isolated from milk-supplemented cultures of *Cerrena unicolor* and *Pycnoporus sanguineus* on colon cancer cells. *3 Biotech* **2021**, *11*, doi:10.1007/s13205-020-02591-w.

104. Liu, K.; Wang, J.; Zhao, L.; Wang, Q. Anticancer, antioxidant and antibiotic activities of mushroom *Ramaria flava*. *Food Chem. Toxicol.* **2013**, *58*, 375–380, doi:10.1016/j.fct.2013.05.001.

105. Khatua, S.; Chandra, S.; Acharya, K. Expanding knowledge on R*ussula alatoreticula*, a novel mushroom from tribal cuisine, with chemical and pharmaceutical relevance. *Cytotechnology* **2019**, *71*, 245–259, doi:10.1007/s10616-018-0280-y.

106. Mansour, A.; Daba, A.; Baddour, N.; El-Saadani, M.; Aleem, E. Schizophyllan inhibits the development of mammary and hepatic carcinomas induced by 7,12 dimethylbenz(α)anthracene and decreases cell proliferation: comparison with tamoxifen. *J. Cancer Res. Clin. Oncol.* **2012**, *138*, 1579–1596, doi:10.1007/S00432-012-1224-0.

107. Norikura, T.; Fujiwara, K.; Narita, T.; Yamaguchi, S.; Morinaga, Y.; Iwai, K.; Matsue, H. Anticancer activities of thelephantin O and vialinin A isolated from *Thelephora aurantiotincta*. *J. Agric. Food Chem.* **2011**, *59*, 6974–6979, doi:10.1021/jf200461j.

108. Chien, R.C.; Lin, Y.C.; Mau, J.L. Apoptotic effect of Taiwanofungus salmoneus (Agaricomycetes) mycelia and solid-state fermented products on cancer cells. *Int. J. Med. Mushrooms* **2017**, *19*, 777–795, doi:10.1615/IntJMedMushrooms.2017024264.

109. Li, M.; Zhang, G.; Wang, H.; Ng, T. Purification and characterization of a laccase from the edible wild mushroom *Tricholoma mongolicum*. *J. Microbiol. Biotechnol.* **2010**, *20*, 1069–1076,

doi:10.4014/JMB.0912.12033.

110. Quang, D.N.; Lam, D.M.; Hanh, N.T.H.; Queb, D.D. Cytotoxic constituents from the fungus *Daldinia concentri*ca (Xylariaceae). *Nat. Prod. Res.* **2013**, *27*, 486–490.

111. Jiang, J.; Sliva, D. Novel medicinal mushroom blend suppresses growth and invasiveness of human breast cancer cells. *Int. J. Oncol.* **2010**, *37*, doi:10.3892/ijo_00000806.

112. Takaku, T.; Kimura, Y.; Okuda, H. Isolation of an antitumor compound from *Agaricus blazei* Murill and its mechanism of action. *J. Nutr.* **2001**, *131*, 1409–1413, doi:10.1093/jn/131.5.1409.

113. Fujimiya, Y.; Suzuki, Y.; Oshiman, K.I.; Kobori, H.; Moriguchi, K.; Nakashima, H.; Matumoto, Y.; Takahara, S.; Ebina, T.; Katakura, R. Selective tumoricidal effect of soluble proteoglucan extracted from the basidiomycete, *Agaricus blazei* Murill, mediated via natural killer cell activation and apoptosis. *Cancer Immunol. Immunother.* **1998**, *46*, 147–159, doi:10.1007/s002620050473.

114. Y Fujimiya; Y Suzuki; R Katakura; T Ebina Tumor-specific cytocidal and immunopotentiating effects of relatively low molecular weight products derived from the basidiomycete, *Agaricus blazei* Murill.. *Anticancer Res .* **1999**, *19*, 113–118.

115. Oshiman, K.I.; Fujimiya, Y.; Ebina, T.; Suzuki, I.; Noji, M. Orally administered β-1,6-D-polyglucose extracted from *Agaricus blazei* results in tumor regression in tumor-bearing mice. *Planta Med.* **2002**, *68*, 610–614, doi:10.1055/s-2002-32904.

116. Rao, Y.K.; Wu, A.T.H.; Geethangili, M.; Huang, M. Te; Chao, W.J.; Wu, C.H.; Deng, W.P.; Yeh, C.T.; Tzeng, Y.M. Identification of antrocin from *Antrodia camphorata* as a selective and novel class of small molecule inhibitor of Akt/mTOR signaling in metastatic breast cancer MDA-MB-231 cells. *Chem. Res. Toxicol.* **2011**, *24*, 238–245, doi:10.1021/tx100318m.

117. Kumar, K.J.S.; Vani, M.G.; Hsieh, H.W.; Lin, C.C.; Wang, S.Y. Antcin-A modulates epithelial-to-mesenchymal transition and inhibits migratory and invasive potentials of human breast cancer cells via p53-mediated mir-200c activation. *Planta Med.* **2019**, *85*, 755–765, doi:10.1055/a-0942-2087.

118. Kowalczewska, M.; Piotrowski, J.; Jedrzejewski, T.; Kozak, W. Polysaccharide peptides from *Coriolus versicolor* exert differential immunomodulatory effects on blood lymphocytes and breast cancer cell line MCF-7 in vitro. *Immunol. Lett.* **2016**, *174*, 37–44, doi:10.1016/j.imlet.2016.04.010.

119. Yoshikawa N; Kunitomo M; Kagota S; Shinozuka K; Nakamura K. Inhibitory effect of cordycepin on hematogenic metastasis of B16-F1 mouse melanoma cells accelerated by adenosine-5′-diphosphate. *Anticancer Res.* **2009**, *29*, 3857–3860.

120. Wang, Z.; Wu, X.; Liang, Y.N.; Wang, L.; Song, Z.X.; Liu, J.L.; Tang, Z.S. Cordycepin induces apoptosis and inhibits proliferation of human lung cancer cell line H1975 via inhibiting the phosphorylation of EGFR. *Molecules* **2016**, *21*, doi:10.3390/molecules21101267.

121. Wei, C.; Yao, X.; Jiang, Z.; Wang, Y.; Zhang, D.; Chen, X.; Fan, X.; Xie, C.; Cheng, J.; Fu, J.; et al. Cordycepin Inhibits drug-resistance non-small cell lung cancer progression by activating AMPK signaling pathway. *Pharmacol. Res.* **2019**, *144*, 79–89, doi:10.1016/j.phrs.2019.03.011.

122. Chang, M.M.; Hong, S.Y.; Yang, S.H.; Wu, C.C.; Wang, C.Y.; Huang, B.M. Anti-cancer effect of cordycepin on FGF9-induced testicular tumorigenesis. *Int. J. Mol. Sci.* **2020**, *21*, 1–21, doi:10.3390/ijms21218336.

123. Martínez-Montemayor, M.M.; Ling, T.; Suárez-Arroyo, I.J.; Ortiz-Soto, G.; Santiago-Negrón, C.L.; Lacourt-Ventura, M.Y.; Valentín-Acevedo, A.; Lang, W.H.; Rivas, F. Identification of biologically active ganoderma lucidum compounds and synthesis of improved derivatives that confer anti-cancer activities in vitro. *Front. Pharmacol.* **2019**, *10*, doi:10.3389/fphar.2019.00115.

124. Wu, G.S.; Lu, J.J.; Guo, J.J.; Li, Y.B.; Tan, W.; Dang, Y.Y.; Zhong, Z.F.; Xu, Z.T.; Chen, X.P.; Wang, Y.T. Ganoderic acid DM, a natural triterpenoid, induces DNA damage, G1 cell cycle arrest and apoptosis in human breast cancer cells. *Fitoterapia* **2012**, *83*, 408–414, doi:10.1016/j.fitote.2011.12.004.

125. Li, F.; Wang, Y.; Wang, X.; Li, J.; Cui, H.; Niu, M. Ganoderic acids suppress growth and angiogenesis by modulating the NF-κB signaling pathway in breast cancer cells. *Int. J. Clin. Pharmacol. Ther.* **2012**, *50*, 712–721, doi:10.5414/CP201663.

126. Yang, Y.; Zhou, H.; Liu, W.; Wu, J.; Yue, X.; Wang, J.; Quan, L.; Liu, H.; Guo, L.; Wang, Z.; et al. Ganoderic acid a exerts antitumor activity against MDA-MB-231 human breast cancer cells by inhibiting the Janus kinase 2/signal transducer and activator of transcription 3 signaling pathway. *Oncol. Lett.* **2018**, *16*, 6515–6521, doi:10.3892/ol.2018.9475.

127. Kodama, N.; Komuta, K.; Nanba, H. Effect of maitake (*Grifola frondosa*) D-Fraction on the activation of NK Cells in cancer patients. *J. Med. Food* **2003**, *6*, 371–377, doi:10.1089/109662003772519949.

128. Masuda, Y.; Nawa, D.; Nakayama, Y.; Konishi, M.; Nanba, H. Soluble β-glucan from *Grifola frondosa* induces tumor regression in synergy with TLR9 agonist via dendritic cell-mediated immunity . *J. Leukoc. Biol.* **2015**, *98*, 1015–1025, doi:10.1189/jlb.1a0814-415rr.

129. Kodama N; Asakawa A; Inui A; Masuda Y; Nanba H. Enhancement of cytotoxicity of NK cells by D-Fraction, a polysaccharide from *Grifola frondosa*. *Oncol Rep.* **2005**, *13*, 497–502.

130. Masuda, Y.; Nakayama, Y.; Mukae, T.; Tanaka, A.; Naito, K.; Konishi, M. Maturation of dendritic cells by maitake α-glucan enhances anti-cancer effect of dendritic cell vaccination. *Int. Immunopharmacol.* **2019**, *67*, 408–416, doi:10.1016/j.intimp.2018.12.039.

131. Masuda, Y.; Nakayama, Y.; Tanaka, A.; Naito, K.; Konishi, M. Antitumor activity of orally administered maitake α-glucan by stimulating antitumor immune response in murine tumor. *PLoS One* **2017**, *12*, doi:10.1371/journal.pone.0173621.

132. Masuda, Y.; Inoue, H.; Ohta, H.; Miyake, A.; Konishi, M.; Nanba, H. Oral administration of soluble β-glucans extracted from *Grifola frondosa* induces systemic antitumor immune response and decreases immunosuppression in tumor-bearing mice. *Int. J. Cancer* **2013**, *133*, 108–119, doi:10.1002/ijc.27999.

133. Masuda, Y.; Togo, T.; Mizuno, S.; Konishi, M.; Nanba, H. Soluble β-glucan from *Grifola frondosa* induces proliferation and Dectin-1/Syk signaling in resident macrophages via the GM-CSF autocrine pathway. *J. Leukoc. Biol.* **2012**, *91*, 547–556, doi:10.1189/jlb.0711386.

134. Masuda, Y.; Ito, K.; Konishi, M.; Nanba, H. A polysaccharide extracted from *Grifola frondosa* enhances the anti-tumor activity of bone marrow-derived dendritic cell-based immunotherapy against murine colon cancer. *Cancer Immunol. Immunother.* **2010**, *59*, 1531–1541, doi:10.1007/s00262-010-0880-7.

135. Kodama, N.; Komuta, K.; Sakai, N.; Nanba, H. Effects of D-Fraction, a polysaccharide from *Grifola frondosa* on tumor growth involve activation of NK cells. *Biol. Pharm. Bull.* **2002**, *25*, 1647–1650, doi:10.1248/bpb.25.1647.

136. Kang, J.H.; Jang, J.E.; Mishra, S.K.; Lee, H.J.; Nho, C.W.; Shin, D.; Jin, M.; Kim, M.K.; Choi, C.; Oh, S.H. Ergosterol peroxide from Chaga mushroom (*Inonotus obliquus*) exhibits anti-cancer activity by down-regulation of the β-catenin pathway in colorectal cancer. *J. Ethnopharmacol.* **2015**, *173*, 303–312, doi:10.1016/j.jep.2015.07.030.

137. He, J.B.; Tao, J.; Miao, X.S.; Bu, W.; Zhang, S.; Dong, Z.J.; Li, Z.H.; Feng, T.; Liu, J.K. Seven new drimane-type sesquiterpenoids from cultures of fungus *Laetiporus sulphureus*. *Fitoterapia* **2015**, *102*, 1–6, doi:10.1016/j.fitote.2015.01.022.

138. Jeff, I.B.; Fan, E.; Tian, M.; Song, C.; Yan, J.; Zhou, Y. In vivo anticancer and immunomodulating activities of mannogalactoglucan-type polysaccharides from *Lentinus edodes* (Berkeley) singer. *Cent. Eur. J. Immunol.* **2016**, *41*, 47–53, doi:10.5114/ceji.2015.56962.

139. Jeff, I.B.; Li, S.; Peng, X.; Kassim, R.M.R.; Liu, B.; Zhou, Y. Purification, structural elucidation and antitumor activity of a novel mannogalactoglucan from the fruiting bodies of *Lentinus edodes*. *Fitoterapia* **2013**, *84*, 338–346, doi:10.1016/j.fitote.2012.12.008.

140. Erkel, G.; Anke, T.; Sterner, O. Inhibition of NF-κB activation by panepoxydone. *Biochem. Biophys. Res. Commun.* **1996**, *226*, 214–221, doi:10.1006/bbrc.1996.1335.

141. Arora, R.; Yates, C.; Gary, B.D.; McClellan, S.; Tan, M.; Xi, Y.; Reed, E.; Piazza, G.A.; Owen, L.B.; Dean-Colomb, W. Panepoxydone targets NF-kB and FOXM1 to inhibit proliferation, induce apoptosis and reverse epithelial to mesenchymal transition in breast cancer. *PLoS One* **2014**, *9*, doi:10.1371/journal.pone.0098370.

142. Fangkrathok, N.; Sripanidkulchai, B.; Umehara, K.; Noguchi H. Bioactive ergostanoids and a new polyhydroxyoctane from *Lentinus polychrous* mycelia and their inhibitory effects on E2-

enhanced cell proliferation of T47D cells. *Nat. Prod. Res.* **2013**, *27*, 1611–1619, doi:10.1080/14786419.2012.742079.

143. Song, K.S.; Li, G.; Kim, J.S.; Jing, K.; Kim, T.D.; Kim, J.P.; Seo, S.B.; Yoo, J.K.; Park, H.D.; Hwang, B.D.; Lim, K.; Yoon, W.H. Protein-bound polysaccharide from *Phellinus linteus* inhibits tumor growth, invasion, and angiogenesis and alters Wnt/β-catenin in SW480 human colon cancer cells. *BMC Cancer* **2011**, *11*, doi:10.1186/1471-2407-11-307.

144. Mei, Y.; Zhu, H.; Hu, Q.; Liu, Y.; Zhao, S.; Peng, N.; Liang, Y. Y. A novel polysaccharide from mycelia of cultured *Phellinus linteus* displays antitumor activity through apoptosis. *Carbohydr. Polym.* **2015**, *124*, 90–97, doi:10.1016/J.CARBPOL.2015.02.009.

145. Lu, T.L.; Huang, G.J.; Lu, T.J.; Wu, J.B.; Wu, C.H.; Yang, T.C.; Iizuka, A.; Chen, Y.F. Hispolon from *Phellinus linteus* has antiproliferative effects via MDM2-recruited ERK1/2 activity in breast and bladder cancer cells. *Food Chem. Toxicol.* **2009**, *47*, 2013–2021, doi:10.1016/J.FCT.2009.05.023.

146. Zhang, G.Q.; Sun, J.; Wang, H.X.; Ng, T.B. A novel lectin with antiproliferative activity from the medicinal mushroom *Pholiota adiposa*. *Acta Biochim Pol .* **2009**, *56*, 415–421.

147. Jagadeesh, R.; Babu, G.; Lakshmanan, H.; Oh, O.M.-J.; Jang, J.K.-Y.; Kong, K.W.-S.; Raaman, N. Bioactive sterol derivatives isolated from the *Pleurotus djamor* var. *roseus* Induced apoptosis in cancer cell lines. *Cardiovasc. Hematol. Agents Med. Chem.* **2020**, *18*, 124–134, doi:10.2174/1871525718666200303123557.

148. Finimundy, T.C.; Abreu, R.M.V.; Bonetto, N.; Scariot, F.J.; Dillon, A.J.P.; Echeverrigaray, S.; Barros, L.; Ferreira, I.C.F.R.; Henriques, J.A.P.; Roesch-Ely, M. Apoptosis induction by *Pleurotus sajor-caju* (Fr.) Singer extracts on colorectal cancer cell lines. *Food Chem. Toxicol.* **2018**, *112*, 383–392, doi:10.1016/j.fct.2018.01.015.

149. Kobori, M.; Yoshida, M.; Ohnishi-Kameyama, M.; Shinmoto, H. Ergosterol peroxide from an edible mushroom suppresses inflammatory responses in RAW264.7 macrophages and growth of HT29 colon adenocarcinoma cells. *Br. J. Pharmacol.* **2007**, *150*, 209–219, doi:10.1038/sj.bjp.0706972.

150. Norikura, T.; Fujiwara, K.; Yanai, T.; Sano, Y.; Sato, T.; Tsunoda, T.; Kushibe, K.; Todate, A.; Morinaga, Y.; Iwai, K.; et al. P-terphenyl derivatives from the mushroom *Thelephora aurantiotincta* suppress the proliferation of human hepatocellular carcinoma cells via iron chelation. *J. Agric. Food Chem.* **2013**, *61*, 1258–1264, doi:10.1021/jf3041098.

151. Wang, H.X.; Liu, W.K.; Ng, T.B.; Ooi, V.E.C.; Chang, S.T. The immunomodulatory and antitumor activities of lectins from the mushroom *Tricholoma mongolicum*. *Immunopharmacology* **1996**, *31*, 205–211, doi:10.1016/0162-3109(95)00049-6.

152. Jeong, J.W.; Lee, W.S.; Go, S.I.; Nagappan, A.; Baek, J.Y.; Lee, J.D.; Lee, S.J.; Park, C.; Kim, G.Y.; Kim, H.J.; Kim, G.S.; Kwon, T.K.; Ryu, C.H.; Shin, S.C.; Choi, Y.H.; Pachymic acid induces apoptosis of ej bladder cancer cells by DR5 up-regulation, ROS generation, modulation of Bcl-2 and IAP family members. *Phytother. Res.* **2015**, *29*, 1516–1524, doi:10.1002/PTR.5402.

153. Ma, J.; Liu, J.; Lu, C.; Cai, D. Pachymic acid induces apoptosis via activating ROS-dependent JNK and ER stress pathways in lung cancer cells. *Cancer Cell Int.* **2015**, *15*, doi:10.1186/S12935-015-0230-0.

154. Jiang, Y.; Fan, L. Evaluation of anticancer activities of Poria cocos ethanol extract in breast cancer: In vivo and in vitro, identification and mechanism. *J. Ethnopharmacol.* **2020**, *257*, doi:10.1016/J.JEP.2020.112851.
